# Role of Acids in Stabilizing Reverse Micelles: Insights from Dodecyl Sulfate

**DOI:** 10.1021/acs.langmuir.6c00158

**Published:** 2026-06-10

**Authors:** Qixuan Li, Marialore Sulpizi

**Affiliations:** Faculty of Physics and Astronomy, Ruhr-University Bochum, Universitätstrasse 150, 44780 Bochum, Germany

## Abstract

The anionic surfactant sodium dodecyl sulfate (SDS) forms reverse micelles (RMs) in two nonmiscible components above the critical micelle concentration (CMC). Although the RMs in salt or alkali solution have been investigated in previous studies, less is known on the working mechanism of acids in SDS RMs. Here, we employ all-atom molecular dynamics simulations with the Generalized Amber Force Field to elucidate how fluoroboric (HBF_4_), chloroauric (HAuCl_4_), phosphoric (H_3_PO_4_), and sulfuric (H_2_SO_4_) acids modulate the interfacial organization, electrostatics, and morphology of SDS-based RMs in toluene. Acid addition alters interfacial dynamics and flexibility by reducing SDS headgroup mobility and tail ordering through persistent hydrogen-bond networks. In SDS bilayer models, acids affect the interfacial tension (γ) in the order γ­(HBF_4_) > γ­(H_2_SO_4_) > γ­(HAuCl_4_) > γ­(H_3_PO_4_). These changes reduce micellar wall flexibility and interfacial tension, potentially promoting the formation of larger and more stable reverse micelles. These insights offer molecular guidelines for tuning RM-mediated nanoparticle synthesis, where subtle interfacial effects dictate nucleation, growth, and structural control.

## Graphical abstract

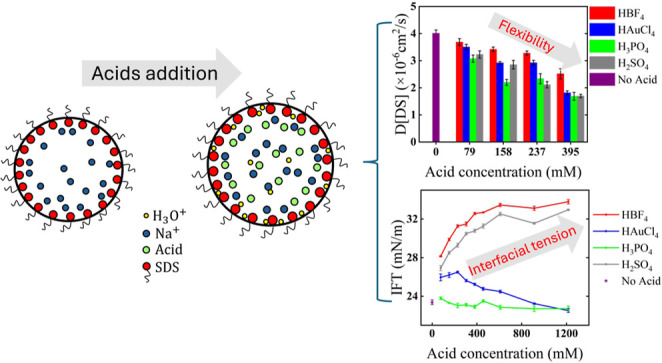

## Introduction

Although normal micelles formed by various ionic or block copolymer surfactants have been widely investigated by experiments^1–10^ and simulations,^11–17^ not so many studies, instead, address reverse micelles (RMs). RMs have applications in diverse fields including enzyme purification, protein encapsulation, ligandability assessment, and nanoparticle synthesis.^18–24^ More specifically, RMs formed by the anionic surfactant dioctyl sodium sulfosuccinate (AOT) and poly­(styrene-*b*-2-vinylpyridine) (PS-*b*-P2VP) block copolymer have been used as nanoreactors for the synthesis of gold (Au) nanoparticles.^25,26^ Here, the micelle core is loaded with the gold precursors ( ${\left[{AuCl}_{4}\right]}^{-}$ ) which, after the addition of a proper reducing agent, are reduced to metallic Au(0), assembling into Au nanoparticles. Importantly, the use of additional reagents can improve the synthesis process controlling the size and the morphology of the RMs. Thus, it is crucial to determine which kind of reagent performs better during the formation of RMs.

Compared to normal micelle, RMs have quite different properties. The stability, morphology, rheology, and size depend on many variables, including the pH, catalyst, surfactant or electrolyte concentration, water-to-surfactant molar ratio (*w*_o_), types of electrolytes, and others.^27–34^ Small-angle X-ray scattering (SAXS) has been used to analyze the structure, size, and rigidity of reverse micelles formed with different surfactants. It has been shown^27,28^ that 1-butanol, used as a cosurfactant, affects the charge present in the ionic micellar interface, which is important to control the size and shape of RMs. Ridley et al.^29,30^ investigated the impact of salt on size and the destabilization in Na-AOT RMs, for which they developed a model to describe the micellar substructure and a predictive equation for the size of the micelle. Sethi et al.^31,32^ reported that *w*_o_ can control the cetyltrimethylammonium bromide (CTAB) reverse micellar structural change, and they designed a hydrotrope-based reverse micellar system to modulate the formation of self-assembled structures. Marques et al.^33^ suggested the headgroup of the ionic reverse micellar surfactants is the dominant variable defining the pH of the water core, which can affect the stability of encapsulated proteins. Abraham et al.^34^ found that increasing the catalyst concentration can enhance the repulsion between the nanoparticle core layer and the RM headgroup layer, leading to an increase in the average hydrodynamic diameter of anionic RMs.

Sodium dodecyl sulfate (SDS), an anionic surfactant which consists of a hydrophobic tail (nonpolar dodecyl group) and a hydrophilic head (polar sulfate group), is investigated and employed for synthesizing the micellar structure. SDS normal micelles have been investigated by all-atom (AA)^35–40^ and coarse-grained (CG)^41–45^ molecular dynamics (MD) simulations. MD simulations also explored the interaction of SDS micelles with proteins, revealing that SDS can act as a denaturant, disrupting protein structure by forming protein-decorated micelles.^35^ Peroukidis et al.^41^ presented a new computational approach for the quantitative prediction of SDS normal micelle formation in dilute aqueous solutions with the modified Martini force field. Wen et al.^45^ computed the scission free energies and the escape free energies of normal micelles to elucidate the transition between spherical and cylindrical morphologies. In the case of SDS, the stabilization of microemulsions with reverse micellar water-in-oil droplets requires the addition of a third component, or cosurfactant, capable of reducing the anionic head repulsion. It has been shown that in the ternary system water/SDS/pentanol a well-defined large phase region of reverse water-in-oil microemulsion droplets is observed with a 1:1 pentanol/toluene mixture.^46,47^ Also, the presence of poly­(diallyldimethylammonium chloride) (PDADMAC) shows a more rigid and ordered surfactant wall of the reverse SDS-based microemulsions in a mixed toluene/pentanol solvent.^48^ In a recent work, Jaugstetter et al.^49^ used SDS surfactants in toluene solutions to establish chloroauric acid-loaded SDS RMs, which can serve as nanoreactors for the synthesis of monodisperse Au nanoparticles on Si substrates. They further demonstrated that AuCl_4_^–^ preferentially accumulates at the micellar interface, which could stabilize the RM structure. These findings highlight the importance of understanding how acids influence the structural and physicochemical properties of SDS RMs. Nowadays, MD simulations have emerged as a valuable tool to provide molecular insights into the (co)­assembly process at a spatiotemporal scale inaccessible to experiments. In our study, we employ AA MD to study the interfacial properties of SDS RMs in water–toluene solutions. Four different acids, fluoroboric acid (HBF_4_), chloroauric acid (HAuCl_4_), phosphoric acid (H_3_PO_4_), and sulfuric acid (H_2_SO_4_), are separately added to the initial aqueous solution. The RMs spontaneously self-assemble in the simulations on a time scale of 10 ns. Our work discusses the distribution of acidic species in the RMs and how this affects the RM interfacial properties, resulting in different size, stability, and rheology.

## Methods

### Simulation Details

All-atom molecular dynamics (AA MD) simulations were performed using the GROMACS 2022.1 software package.^50^ The generalized Amber force field (GAFF)^51^ was employed to describe atomistic interactions, with water molecules modeled using the SPC model.^52^ To improve simulation accuracy for SDS surfactants, we specifically applied the amberlipid 14 force field,^53^ an extension of GAFF.

The acidic species were modeled according to their predominant dissociation in solution. HBF_4_,^54^ HAuCl_4_,^55^ and H_2_SO_4_ ^56^ are strong acids with very low p*K*_a_ values and were considered fully dissociated. H_3_PO_4_ ^57^ has three dissociation steps, with the first p*K*_a_ significantly lower than the subsequent steps, indicating that it primarily exists as H_2_PO_4_^–^ and H^+^ under standard conditions. However, at high concentrations, deprotonation of H_3_PO_4_ can be suppressed, and most molecules remain undissociated. Accordingly, the acidic environments were represented using the fully dissociated anions (BF_4_^–^, AuCl_4_^–^, and HSO_4_^–^) and hydrated protons (H_3_O^+^) for HBF_4_, HAuCl_4_, and H_2_SO_4_ and undissociated H_3_PO_4_.

The initial structures of the acidic anions were optimized at the B3LYP-D3/def2-TZVP level of theory.^58^ Cartesian Hessian matrices were subsequently generated using wB97M-V/def2-TZVP in ORCA.^59^ Topology files were prepared with Sobtop (version 1.0­(dev5), Tian Lu, https://sobereva.com/soft/Sobtop, accessed 22-Nov-2024) using the m2seminario method, and RESP charges were assigned with Multiwfn 3.8.^60,61^ All species employed in the simulations are shown in Figure 1a.

**Figure 1:**
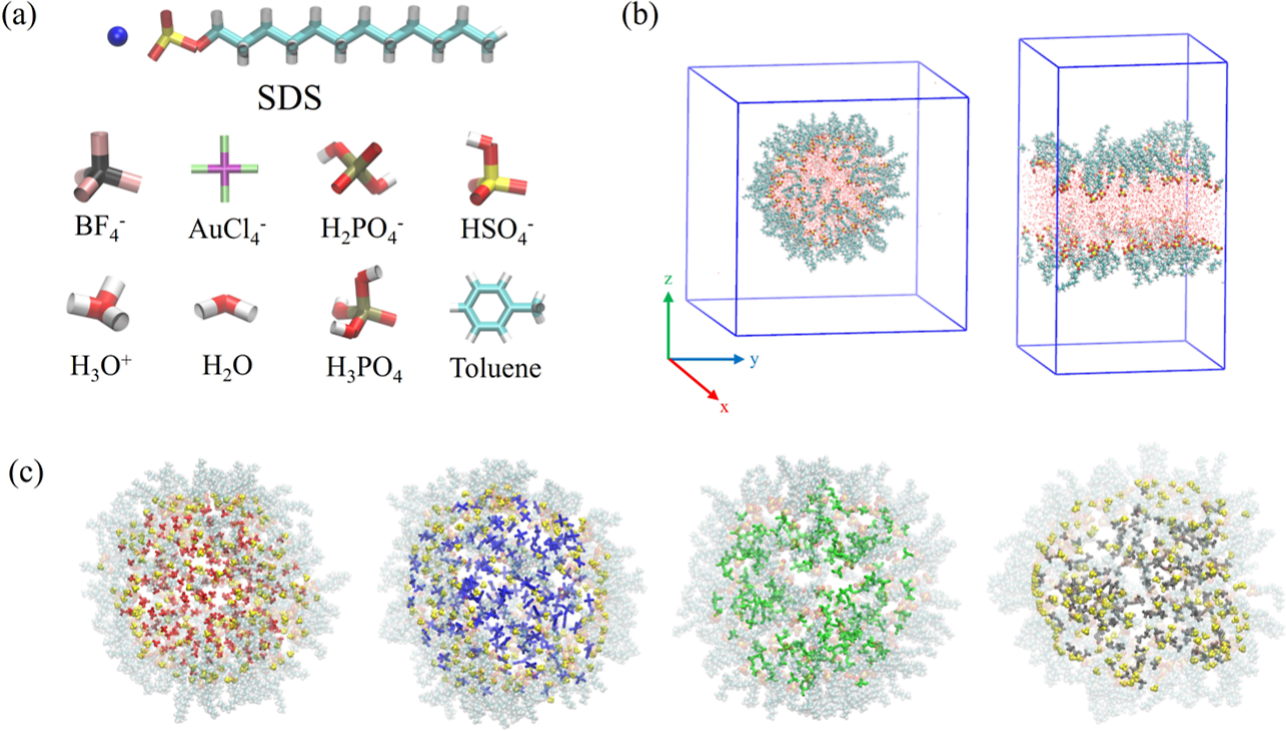
(a) Molecular configurations for all species and molecules in this work. (b) Snapshots of the simulation boxes for the RM_I_ (left) and BI_I_ (right), respectively, while the toluene molecules are not shown for clarity. (c) The last snapshots of RM_I_ included with different acids, respectively. BF_4_^–^, AuCl_4_^–^, H_3_PO_4_, and HSO_4_^–^ are represented by red, blue, green, and gray cubes, respectively, and H_3_O^+^ is represented by yellow beads. The SDS surfactants are shown in transparent beads, and toluene is not shown for clarity.

Accurately measuring the surface tension of spherical RMs is challenging; therefore, SDS bilayers were employed to mimic the local interfacial environments of the RMs, providing insights into interfacial properties under comparable acid concentrations. RMs were constructed at two different water-to-surfactant ratios (*w*_o_) to control their sizes, and analyses were performed across both sizes to minimize the influence of the micellar size on conclusions regarding acid effects. Modeling details are summarized in Table 1. The systems are denoted as RM_I_, RM_II_, BI_I_, and B*I*_II_. RM_I_ and RM_II_ correspond to RMs with *w*_o_ = 25 and *w*_o_ = 40, respectively, while BI_I_ and BI_II_ represent bilayers with comparable interfacial environments to RM_I_ and RM_II_, respectively.

**Table 1:** Molecular Configuration Parameter Setting of This Work^a^

system	box size (nm^3^)	*w*_o_	number of molecules			simulation time (ns)
			SDS	water	toluene	
RM_I_	13 × 13 × 13	25	200	5000	10,000	100
RM_II_	16 × 16 × 16	40	400	16,000	20,000	100
BI_I_	7 × 7 × 12	40	100	4000	2000	100
BI_II_	9 × 9 × 13	50	200	10,000	4000	100

a A smaller reverse micelle (RM_*I*_) and a larger reverse micelle (RM_*II*_) with respect to the similar interfacial environments in SDS bilayers are defined as BI_*I*_ and BI_*II*_, respectively.

Initial molecular configurations for RMs and SDS bilayers were generated using Packmol.^62^ All simulations began with energy minimization via the steepest descent method,^63^ followed by equilibration in the *NPT* ensemble using the V-rescale thermostat^64^ and Berendsen barostat.^65^ Long-range electrostatic interactions were treated with the particle mesh Ewald method,^66^ and a 1.4 nm cutoff was applied for nonbonded interactions.

Production MD simulations employed the V-rescale thermostat to maintain a temperature of 300 K and the Parrinello–Rahman barostat^67^ to maintain a pressure of 1.0 bar, with isotropic coupling for RMs and semiisotropic coupling for SDS bilayers. All hydrogen atom bonds were constrained using the LINCS algorithm.^68^ Analyses were performed on the trajectories from the last 10 ns of each simulation. Simulation snapshots were visualized using VMD,^69^ and representative final configurations of RM_I_ and BI_I_ are shown in Figure 1b.

### Convexity

Convexity (Ξ) is a metric used to quantify surface roughness, defined in terms of the volume and convex hull of the shapes under investigation, with values ranging from 0 < Ξ ≤ 1. A perfect sphere without any surface irregularities corresponds to Ξ = 1, while a value of Ξ approaching 0 indicates increasingly irregular shapes with greater missing volume, although Ξ = 0 is physically unrealistic. This approach has been successfully applied to analyze the convexity of AOT RMs by generating Willard–Chandler surfaces^70^ using the PyTim package,^71^ as demonstrated by Gale et al.^72,73^ In this work, we adapted their Python code to calculate the convexity of SDS RMs from 10 ns trajectories using MDAnalysis^74,75^ and manipulated mesh surfaces with PyVista.^76^

### Tail Order Parameter

Different acids can modulate the interfacial flexibility of SDS surfactant tails. To quantify this, the order parameter^77^ of the SDS tails was calculated. For the *n*th carbon atom in the tail, the order parameter is defined as

1 $$S=\frac{3\left\langle \cos^{2}\theta \right\rangle -1}{2}$$

θ denotes the angle between the vector defined by the (*n* – 1)­th and (*n* + 1)­th carbon atoms and a specific vector. For RMs, the reference vector is defined as the vector connecting the SDS headgroup to the center of mass (COM) of the micelle, whereas for bilayers, it is defined as the *Z*-axis. Accordingly, the order parameters for RMs and bilayers are denoted as *S*_CS_ and *S*_Z_, respectively.

### Interfacial Tension

The interfacial tension (IFT) of the SDS bilayers was calculated from the normal and lateral pressure components according to

2 $$\gamma =\frac{1}{2}\left(P_{zz}-\frac{P_{xx}+P_{yy}}{2}\right)\cdot L_{z}$$

where *L*_*z*_ is the length of the simulation box along the *z*-direction. *P*_*xx*_, *P*_*yy*_, and *P*_*zz*_ are the pressure tensor components in the *x*, *y*, and *z* directions, respectively.

### Diffusion Coefficient

To quantify the interfacial mobility of SDS surfactants, the diffusion coefficient (*D*[*DS*]) was calculated using Einstein’s relation:

3 $$D\left[DS\right]=\lim_{t\to \infty }\frac{\left\langle \sum_{i}^{n}{\left|{\mathrm{r⃗}}_{COM}\left(t\right)-{\mathrm{r⃗}}_{COM}\left(0\right)\right|}^{2}\right\rangle }{6t}$$

where ${\mathrm{r⃗}}_{COM}\left(t\right)$ and ${\mathrm{r⃗}}_{COM}\left(0\right)$ denote the center-of-mass (COM) positions of surfactant *i* at the initial time and after a time interval *t*, respectively. The ensemble-averaged mean square displacement (MSD), $|{\mathrm{r⃗}}_{COM}\left(t\right)-{\mathrm{r⃗}}_{COM}\left(0\right){|}^{2}$ , was computed, and *D*[*DS*] was obtained from the slope of the MSD as a function of time.

### Coordinate-Pair Eccentricity

To characterize the three-dimensional shape of RMs, the coordinate-pair eccentricity (CPE) was calculated using the three semiaxis lengths (*a*, *b*, and *c*), following the approach developed by Gale et al.^73,74^ The CPE is defined as

$$e_{ab}=\sqrt{1-\frac{b^{2}}{a^{2}},}{,e}_{ac}=\sqrt{1-\frac{c^{2}}{a^{2}}}$$ 4

where *e*_ab_ and *e*_ac_ constitute the eccentricity pairs used to describe deviations of the micellar shape from spherical symmetry in three dimensions.

### Acid and Hydrogen-Bond Dynamics

Different acids exhibit different affinities for the SDS headgroups at the RM interface, which are associated with their ability to stabilize the micellar structure. Hydration effects and electrostatic interactions between hydrated protons and the headgroups of anionic RMs are known to influence interfacial behavior.^78–80^ In addition, the long-range polarizability of acidic anions affects their diffusivity in aqueous solution, thereby modulating their association with interfacial headgroups. To quantify these interactions, the distance between the central atoms of the acidic species (B, Au, S, and P) and the center of mass (COM) of all SDS surfactants was calculated. This distance was then used to compute the distance autocorrelation function (*C*_*d*_*t*__):

5 $$C_{d_{t}}=\frac{\sum_{t}^{N-k}\left(d_{t}-\mathrm{d̅}\right)\left(d_{t+k}-\mathrm{d̅}\right)}{\sum_{t}^{N}{\left(d_{t}-\mathrm{d̅}\right)}^{2}}$$

where *d*_*t*_ represents the distance at time *t*, *k* is the tag of time, *N* corresponds to the quantities of *d*_*t*_ in the specific duration, and *d̅* is the mean of the series *d*_*t*_.

Hydrogen-bond (H-bond) dynamics between hydrated protons and SDS headgroups were analyzed using the *gmx hbond* tool with the autocorrelation option. The resulting hydrogen-bond autocorrelation function, *C*_HB_(*t*), was fitted with a biexponential function to quantify H-bond lifetimes

6 $$C_{HB}\left(t\right)=A\cdot e^{-t/\tau_{1}}+B\cdot e^{-t/\tau_{2}}$$

where τ_1_ and τ_2_ correspond to short-time and long-time dynamical components, respectively. The amplitudes *A* and *B* represent the relative contributions of these components and satisfy *A* + *B* = 1. An effective H-bond lifetime, *T*_HB_, is defined as *A*·τ_1_ + *B*·τ_2_, providing a convenient metric for comparing hydrogen-bond dynamics across different systems.

### Electrostatic Potential and Water Orientation

The charge density distribution was used to compute the electrostatic potential for RMs and SDS bilayers. For RMs, a spherical integration was considered based on the CPE analysis. Accordingly, the radial charge density, ρ­(*r*), was evaluated as a function of the distance *r* from the micelle center of mass, including contributions from all surfactants. The electrostatic potential of the RMs, ψ­(*r*), was then obtained by solving the Poisson’s equation:

7 $$\psi \left(r\right)-\psi \left(0\right)=-\frac{1}{\varepsilon_{0}}\int_{0}^{r}{\left(\frac{1}{r^{\prime }}\right)}^{2}dr^{\prime }\int_{0}^{r^{\prime }}{\left(r^{\prime \prime }\right)}^{2}\rho \left(r^{\prime \prime }\right)dr^{\prime \prime }$$

where ρ­(*r*″) denotes the charge density and ε_0_ is the vacuum permittivity. The accumulated charge, *Q*(*r*), is defined as

8 $$Q\left(r\right)-Q\left(0\right)=4\pi \int_{0}^{r}{\left(r^{\prime \prime }\right)}^{2}\rho \left(r^{\prime \prime }\right)dr^{\prime \prime }$$

For the SDS bilayers, the electrostatic potential, ψ­(*z*), according to Poisson’s equation for the slab geometry, reads

9 $$\psi \left(z\right)-\psi \left(0\right)=-\frac{1}{\varepsilon_{0}}\int_{0}^{z}dz^{\prime }\int_{0}^{z^{\prime }}\rho \left(z^{\prime \prime }\right)dz^{\prime \prime }$$

where ρ­(*z*″) is the charge density at position *z*″ and ψ(0) denotes the electrostatic potential at the bilayer midplane (*z* = 0).

The orientation of interfacial water molecules is closely linked to the local electrostatic potential.^81–84^ Water orientation is quantified using the tilt angle (ϕ), defined as the angle between the dipole moment vector of a water molecule and the *z*-axis. The bilayer center was set to *z* = 0, and the average orientation, ⟨cos­(ϕ)⟩, was computed in slabs of 0.1 nm thickness extending to *z* ± 1.5 nm for BI_I_ and to *z* ± 2.0 nm for BI_II_.

### Potential of Mean Force

The potential of mean force (PMF) to evaluate the ion affinity for the micellar wall was calculated by pulling each acid species (BF_4_^–^, AuCl_4_^–^, HSO_4_^–^, or H_3_PO_4_) along the *z*-axis perpendicular to the BI. The reaction coordinate (*Z*) is defined as the vertical distance from the center of mass of acidic species to the center of mass of SDS BIs. The chosen pulling rate is 0.002 nm/ps with a harmonic force constant of 1000 kJ/(mol nm^2^).

To obtain the PMF, we used umbrella sampling simulations.^85^ A total of 100 umbrella windows with a spacing of 0.02 nm are simulated with a 1 ns simulation time for each window. A harmonic force constant of 1000 kJ/(mol nm^2^) is applied for each window. The weighted histogram analysis method (WHAM),^86^ in particular the *gmx wham* tools, was employed to generate the PMFs from the umbrella sampling windows. The error bars of each point in the PMFs are estimated using a bootstrap method.^87^

## Results and Discussion

To investigate the structural and dynamical properties of SDS-based RMs, we considered a series of model systems comprising a smaller RM (RM_I_), a larger RM (RM_II_), and two different SDS bilayers (BIs), referred to as BI_I_ and BI_II_, representing the limiting case of very large RMs (diameter > 20 nm) for which the micellar wall can be approximated as a locally flat bilayer. To the SDS/toluene/water mixtures, we separately added four acids, namely, HBF_4_, HAuCl_4_, H_3_PO_4_, and H_2_SO_4_, to examine their influence on RM properties (Figure 1c). All acids were modeled in their fully dissociated forms (acidic anion plus hydronium), except H_3_PO_4_. This treatment is justified by their *pK*_a_ values: HBF_4_ (*pK*_a_ around −4.9) and HAuCl_4_ (also a strong acid, though with an imprecisely determined *pK*_a_) are expected to be fully dissociated under the conditions studied. H_2_SO_4_, with *pK*_a1_ ≈ −3 and *pK*_a2_ ≈ 2, undergoes complete dissociation in its first ionization step. In contrast, H_3_PO_4_, with *pK*_a1_ ≈ 2.1, *pK*_a2_ ≈ 7.2, and *pK*_a3_ ≈ 12.4, exhibits only limited dissociation at high concentrations. Thus, we used the original nondissociated H_3_PO_4_ model to represent phosphoric acid-loaded RMs.

In the core of the RM, large variations of the pH are not expected in analogy to the AOT micelles,^88–90^ which share similar sulfonic groups as surfactant heads. However, the situation may be different at the micellar wall, where the high ion concentration may indeed shift the acid equilibrium. Estimates of actual p*K*_a_ values in the confined environment are beyond the capability of force field models; however, for a comparison, in the Supporting Information, we also discuss the results for the case of H_3_PO_4_ fully or partially dissociated into H_3_O^+^ and H_2_PO_4_^–^ ((Figures S15–S17)). All systems spontaneously self-assembled from randomly distributed components within 10 ns of simulation time.

### Structural Properties

#### Ion Distribution between the Water Pool and Interfacial Wall

In all investigated systems, the SDS RMs self-assemble in approximately a spherical shape as quantified by the CPE parameter (Table S1). The distribution of different molecular species is analyzed in terms of probability density distributions (*P*(*r*)), which are reported for RM_I_ and RM_II_ in Figures 2 and S1, respectively. In the absence of acid, sodium ions are most likely found in the proximity of the SDS headgroups, in a similar way as experimentally observed for Na-AOT RMs.^91^ However, upon acid addition, both acidic anions and protons accumulate at the micellar interface, altering the spatial organization of the SDS headgroups and associated sodium ions. Proton accumulation at the micellar interface has previously been proposed^88,89,91^ for AOT reverse micelles, whose surfactant headgroups also contain negatively charged sulfonate groups. Measurements of the pH within the water pool indicate that the core region of the reverse micelles remains close to apparent neutral pH, whereas protons preferentially migrate toward the interfacial region. This spatial redistribution of protons has been invoked to rationalize the buffer-like behavior of the confined water pool in AOT reverse micelles.^88–90^ Direct interactions between hydronium ions (H_3_O^+^) and sulfonate (RSO^3–^) groups have also been proposed on the basis of recent vibrational spectroscopic measurements in AOT micelles.^92^ Similar to acidic conditions, alkaline conditions in AOT reverse micelles do not translate directly into a uniformly high internal pH. It was previously shown that the buffering effect of the surfactant headgroups can modify interfacial interactions and water structure to maintain a distinct microenvironment that enhances catalytic activity.^33,93^

**Figure 2:**
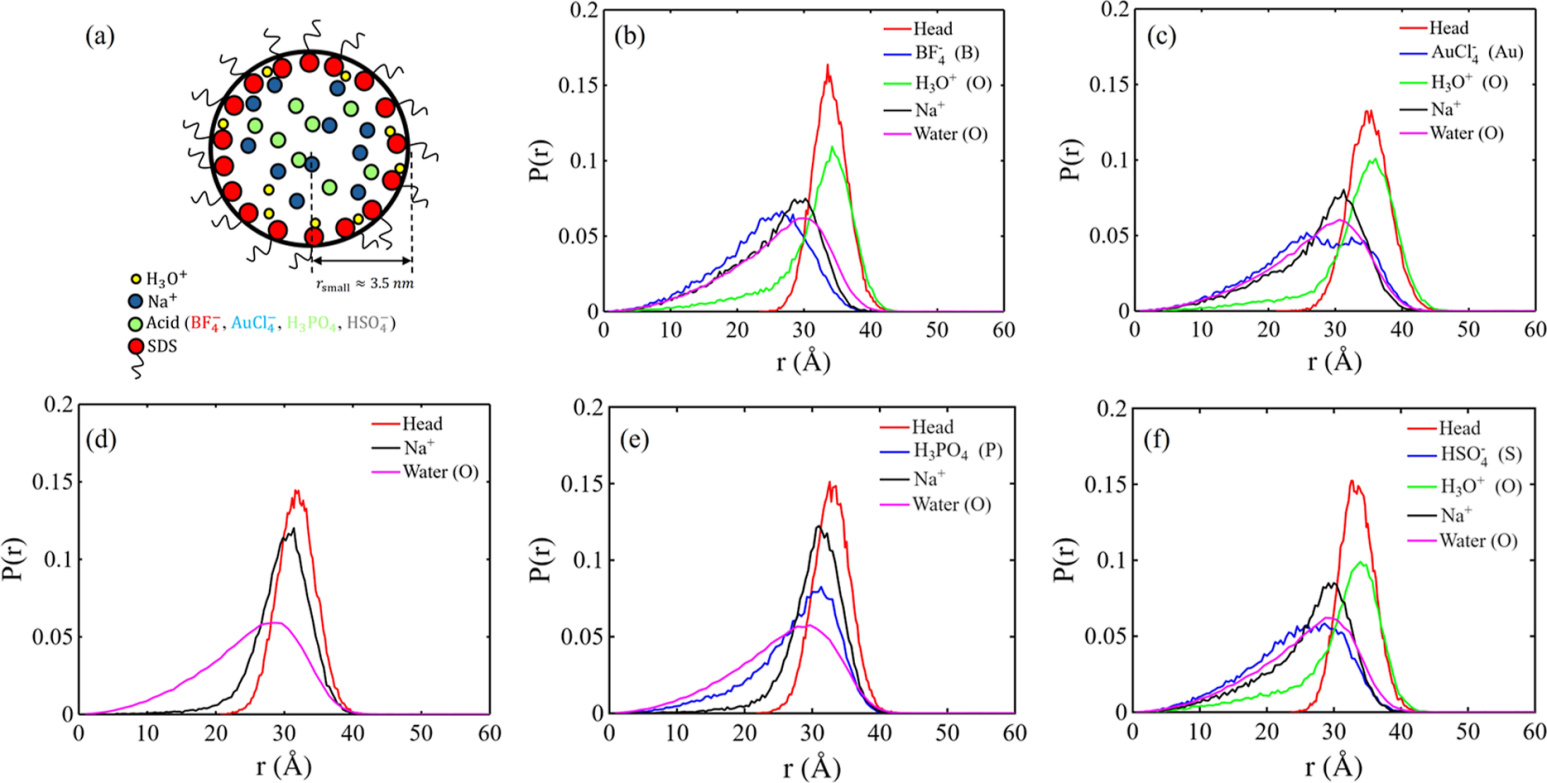
(a) The schematic acidic species distribution inside the RMs in the case of four acids. (b–f) Probability density distribution of the distance (*P*(*r*)) of the specific types of atoms from the center of mass (COM) for all SDS surfactants. (b), (c), (e), and (f) are the RM_I_ with 158 mM HBF_4_, HAuCl_4_, H_3_PO_4_, and H_2_SO_4_, respectively, and (d) represents the RMs without acids.

#### Clear Ion-Specific Effects Emerge

For HBF_4_, HAuCl_4_, and H_2_SO_4_, protons preferentially replace sodium ions to compensate for the negative charge of the SDS headgroups, yielding proton distributions whose maxima coincide with those of the headgroup region. The behavior of the acidic anions displays notable differences. In HAuCl_4_-loaded solutions, AuCl_4_^–^ ions approach the micellar wall more closely than the other anions, resulting in a broader SDS headgroup distribution and a clear layering effect, which is slightly more pronounced in the larger RM_II_ (Figures 2c and S1c). The main reason why AuCl_4_^–^ exhibits stronger adsorption is its higher polarizability and more distributed charges, which render partial dissolvation at the interface less costly. More specifically, as seen in Figure S3b,f, an additional interfacial layer appears between the inner water pool and the hydrophobic boundary. This result is consistent with experimental data,^49,94^ which showed that HAuCl_4_ partitions strongly to micellar interfaces and enhances the stability of both reverse and normal micelles. In contrast, in the case of H_3_PO_4_ solutions, sodium ions remain the predominant counterions at the interface. This behavior is in line with the tendency of H_3_PO_4_ to form oligomeric (H_3_PO_4_)_*n*_ species at high concentrations, as also previously experimentally reported,^95,96^ which diminishes its ability to modulate the interfacial environment. When H_3_PO_4_ adsorbs at the micellar wall (Figure S3c,g), it exhibits a broad and heterogeneous distribution that extends into the bulk water region, effectively leaving interfacial space available for sodium ions (Figure 2e). Similarly, HSO_4_^–^ is known to form (HSO_4_^–^)_*n*_ aggregates in waters.^97–99^ In the H_2_SO_4_-RM system, HSO_4_^–^ anions reside predominantly in the aqueous core rather than at the interface (Figure S3d,h), due to its low polarizability compared to other polyatomic anions and to the electrostatic repulsion from the negatively charged SDS headgroups. To quantify the acidic anion propensity for the interfacial wall, the potential of mean force (PMF) profiles have been calculated in the case of high dilution (2.6 mM) for the bilayer system and are reported in the section “Calculation of the potential of mean force for the different acidic species” of the Supporting Information. As shown in Figure S14, AuCl_4_^–^ and H_3_PO_4_ exhibit some affinity for the interface, which is stronger for H_3_PO_4_, while BF_4_^–^ and HSO_4_^–^ prefer to reside in bulk. This is in agreement with the analysis of the ion distributions at higher concentrations. As previously stressed, the higher affinity of AuCl_4_^–^ for the micellar wall with respect to BF_4_^–^ and HSO_4_^–^ is due to its higher polarizability and more distributed charges. In the case of H_3_PO_4_, the even higher affinity for the micellar wall is due to the overall neutral charge.

#### Interfacial Thickness and Electrostatic Properties

The different propensities of the ions to populate the micellar interface also modulate the interfacial thickness and, in turn, the interface potential. H_3_PO_4_-loaded RMs show a substantially thicker interfacial region compared to the other cases (Figure S4c,g). Given the generally inverse relationship between interfacial thickness and interfacial tension,^100^ we explore the implications for interfacial tension in a subsequent section.

The specific ion distribution modulates the electrostatic potential across the RMs, modifying in turn the RM aggregation behavior. The balance between the repulsive forces (due to the surface potential) and attractive forces (hydrophobic interactions) determines the final size and shape of the micelle. Stronger electrostatic repulsion tends to favor smaller, more spherical micelles, as it limits how closely the headgroups can pack. Reducing the potential difference, by increasing the ionic strength or varying the pH, allows for tighter packing and can cause the micelles to grow larger and transition to different shapes (e.g., ellipsoidal or cylindrical).

The electrostatic potential profile for the RMs (ψ­(*r*)) and for the BIs (ψ­(*z*)) has been calculated, setting as zero reference the value in the aqueous water solution (Figures 3a,b and S5a,b). The maximum in each profile (Ψ_max_) corresponds to the location of the negatively charged SDS headgroups. The highest peak is found in the case of HBF_4_ (for both BIs and RMs), reflecting the weakest compensation of the SDS headgroup charge by hydrated protons (Figure S6a,e). H_2_SO_4_ yields a peak of comparable magnitude, consistent with its similar interfacial ion distribution. In systems loaded with HAuCl_4_, the peak is reduced relative to HBF_4_ and H_2_SO_4_, owing to the closer approach of AuCl_4_^–^ to the interface, which broadens the distribution of interfacial charge. The lowest peak is found for H_3_PO_4_, where extensive accumulation of sodium ions at the micellar wall leads to substantial neutralization of the SDS headgroup charge (Figure S6c,g).

**Figure 3:**
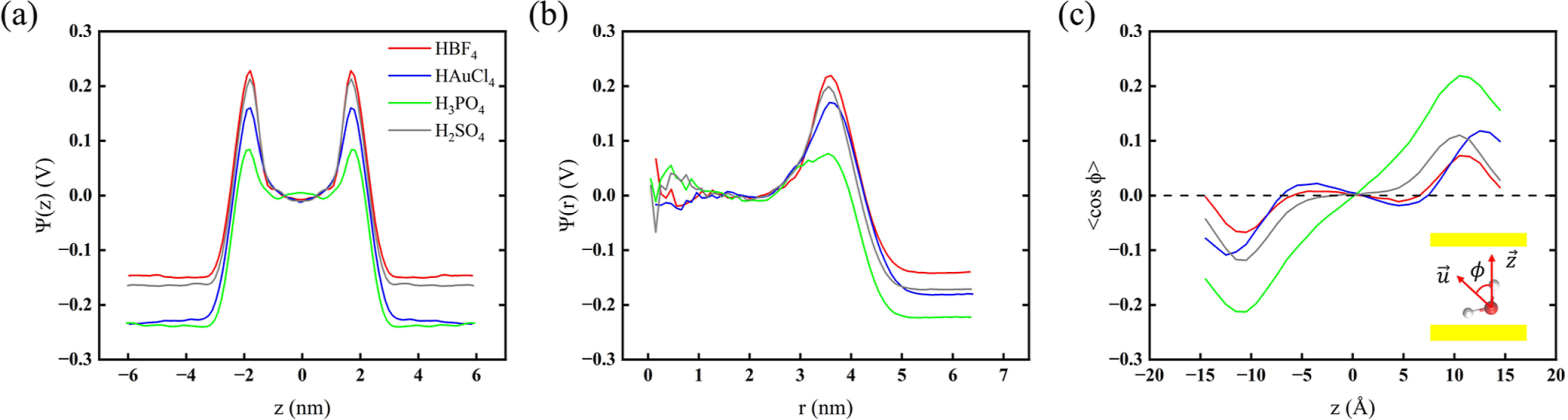
(a,b) Electrostatic potential of BI_I_ (ψ­(*z*)) and BI_I_ (ψ­(*r*)). (c) Water orientation of BI_I_. The tilt angle (ϕ) between the dipole moment vector of a water molecule (u⃗) and the *z*-axis (z⃗) . The acid concentrations used in BI_I_ and RM_I_ are 380 mM and 158 mM, respectively.

As shown in Table 2, the potential difference across the interface, defined as

10 $$\Delta \Psi =\Psi_{AQU}-\Psi_{TOL}$$

follows the trend

$$\Delta \Psi_{\left(HBF_{4}\right)}<\Delta \Psi_{\left(H_{2}SO_{4}\right)}<\Delta \Psi_{\left(HAuCl_{4}\right)}<\Delta \Psi_{\left(H_{3}PO_{4}\right)}$$

indicating that acid identity significantly influences interfacial electrostatic properties. The ability of the acidic species to screen the potential at the micellar wall can be observed on the different water orientations inside the RMs, as reported in Figures 2c and S5c. Water molecules are most strongly ordered in the presence of H_3_PO_4_, exhibiting the largest projection of their dipole moments along the axis normal to the bilayer. In contrast, for the other three acids, water is considerably less ordered: the dipole projection averages to zero across a broader region between the opposing surfactant layers, indicating more effective electrostatic screening and a reduced directional bias.

**Table 2:** Interfacial Thickness (IT) in RMs and BIs, with Units of nm^a^

system	acid concentration	IT	ΔΨ	Ψ_max_
RM_I_	158 mM HBF_4_	1.55	0.14	0.22
RM_I_	158 mM HAuCl_4_	1.60	0.18	0.18
RM_I_	158 mM H_3_PO_4_	1.69	0.22	0.08
RM_I_	158 mM H_2_SO_4_	1.58	0.17	0.20
RM_II_	193 mM HBF_4_	1.58	0.12	0.22
RM_II_	193 mM HAuCl_4_	1.65	0.18	0.16
RM_II_	193 mM H_3_PO_4_	1.67	0.21	0.08
RM_II_	193 mM H_2_SO_4_	1.60	0.13	0.22
BI_I_	380 mM HBF_4_	1.51	0.15	0.23
BI_I_	380 mM HAuCl_4_	1.54	0.23	0.16
BI_I_	380 mM H_3_PO_4_	1.62	0.24	0.08
BI_I_	380 mM H_2_SO_4_	1.52	0.17	0.21
BI_II_	360 mM HBF_4_	1.53	0.14	0.24
BI_II_	360 mM HAuCl_4_	1.57	0.20	0.18
BI_II_	360 mM H_3_PO_4_	1.65	0.23	0.09
BI_II_	360 mM H_2_SO_4_	1.56	0.17	0.22

a The potential difference (ΔΨ) and peaks (Ψ_max_), with units of *V.*

### Interfacial Flexibility

In this section, we discuss the impact of acids on the flexibility of the RM wall. This is a key property affecting, in turn, micelle shape and size, as well as stability. Less flexible walls in micelles can lead to larger micelle sizes and potentially altered viscoelastic properties. This is because the stiffness of the micelle’s “wall” (formed by the surfactant molecules) influences how easily the micelle can bend and change shape.

#### Diffusion of Different Components

First information about micellar wall flexibility can be obtained from the diffusion coefficient of the SDS headgroups (D­[DS], Figures 4a and S7a, for RM_I_ and RM_II_, respectively). In fact, a faster diffusion of surfactant headgroups is generally correlated with higher micellar wall flexibility. *D*[*DS*] decreases with increasing acid concentration, according to the sequence:

$$\begin{array}{ll} D{\left[DS\right]}_{No-Acid} & >D{\left[DS\right]}_{HBF_{4}}>\\ {D[DS]}_{HAuCl_{4}} & >D{\left[DS\right]}_{H_{2}SO_{4}}\approx D{\left[DS\right]}_{H_{3}PO_{4}} \end{array}$$

suggesting that the diffusion dynamics of SDS headgroups is affected by the interaction between acids and surfactants.

**Figure 4:**
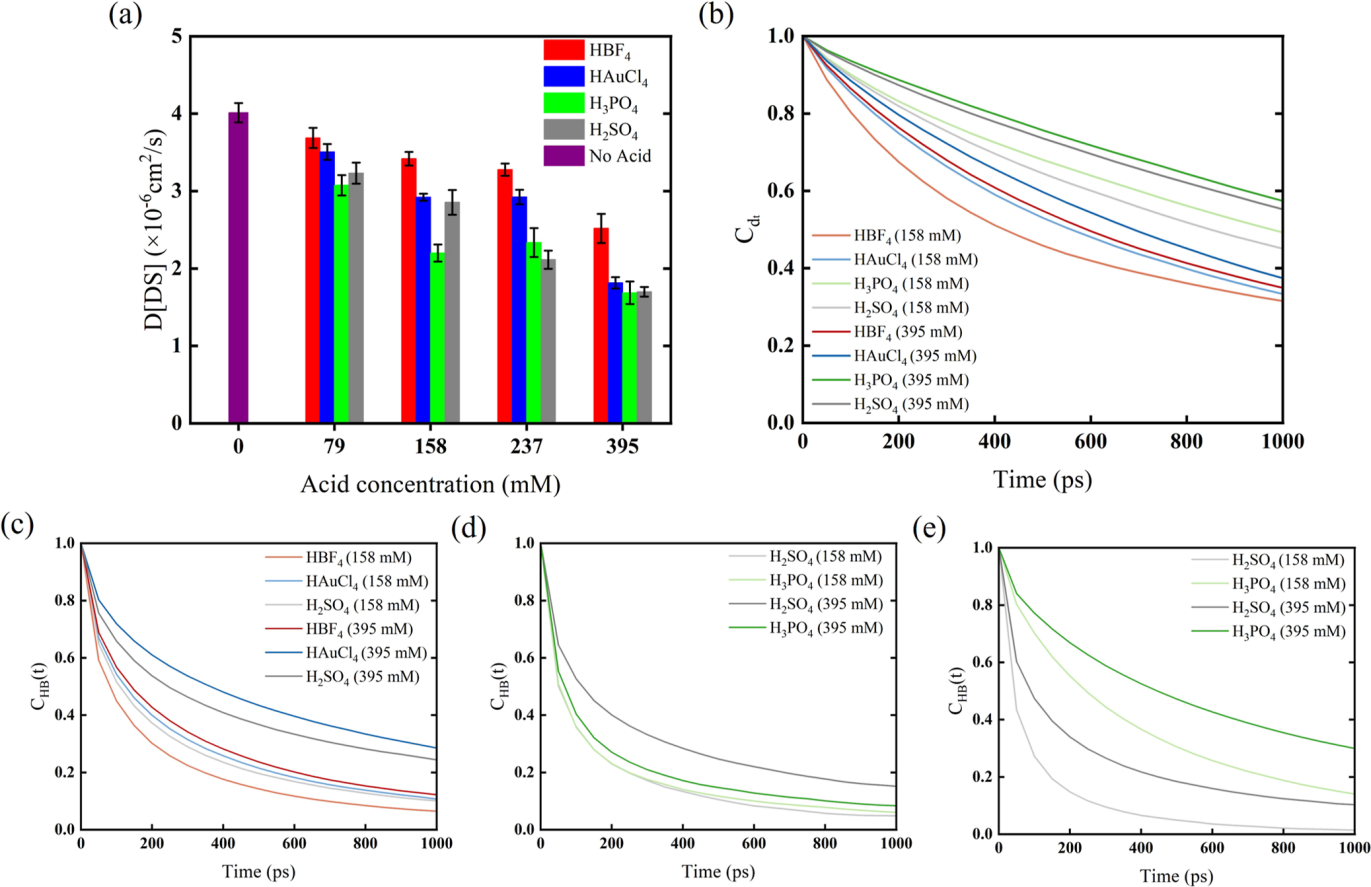
(a) Diffusional coefficient of SDS surfactants (*D*[*DS*]) for RM_I_ with different acid concentrations. (b) Autocorrelation function of distance (*C*_*d*_*t*__): the distance is between selected acidic species and the center of mass (COM) of SDS surfactants. (c–e) Autocorrelation function of hydrogen bonds (H-bonds) (*C*_HB_(*t*)): the H-bond is between hydrated protons and headgroups of SDS surfactants in (c), the H-bond is between hydrated protons and acidic species (HSO_4_^–^ or H_3_PO_4_) in (d), and the H-bond is between acidic species and acidic species (HSO_4_^–^···HSO_4_^–^ or H_3_PO_4_ ··· H_3_PO_4_) in (e). The acid concentrations are 158 mM and 395 mM, respectively.

In Figures 4b and S7, we present the autocorrelation functions of the distance between the acidic anions and the center of mass of the SDS surfactants. These data indicate that BF_4_^–^ diffuses faster than AuCl_4_^–^, consistent with its weaker affinity for the micellar interface and greater hydrophilicity. AuCl_4_^–^ has a layering effect at the interface, which is related to its stronger hydrophobicity and polarizability, which can slightly decrease the interfacial diffusivity compared to BF_4_^–^. In contrast, HSO_4_^–^ and H_3_PO_4_ exhibit markedly slower diffusion due to their ability to form hydrogen bonds with the SDS headgroups, an effect that contributes to enhanced interfacial stability in the RMs.

Given that HBF_4_, HAuCl_4_, and H_2_SO_4_ are modeled as fully dissociated into hydrated protons and their conjugate bases, it is essential to consider the combined influence of both components on interfacial dynamics. As shown in the previous section, hydrated protons preferentially accumulate at the interface, suggesting that the persistence of hydrogen bonds between protons and SDS headgroups may correlate with a reduced surfactant mobility.

In H_2_SO_4_-loaded systems, HSO_4_^–^ forms stable hydrogen bonds not only with the SDS headgroups but also with neighboring HSO_4_^–^ ions or hydronium. A similar behavior is observed for H_3_PO_4_, as reflected in the hydrogen-bond autocorrelation decays in Figure 4d,e. Biexponential fits yield the corresponding hydrogen-bond lifetimes (Table S2 and Figure S8). These extended hydrogen-bond networks linking SDS headgroups with HSO_4_^–^, H_3_O^+^, or H_3_PO_4_ act to stabilize the interfacial layer and suppress surfactant diffusivity, reducing the *D*[*DS*].

Overall, the diffusivities of the acidic species follow the trend HBF_4_ > HAuCl_4_ > H_2_SO_4_ ≈ H_3_PO_4_, a behavior that reflects differences in hydrophilicity, solubility, and hydrogen-bonding capacity at the micellar interface.

Previous studies had shown that the addition of acids can facilitate the formation of normal micelles and stabilize the micellar structure by influencing interfacial interactions. In particular, hydrochloric acid (HCl) and amino acids were shown to reduce the critical micelle concentration (CMC) in SDS solutions by screening electrostatic repulsions between headgroups,^101,102^ thereby enhancing micellar stability. In the subsequent sections, we will discuss how the hydrated protons and acid species affect the headgroups at the interface, indicating their influence on the stabilization of RMs.

#### Acids Reduce the SDS Tail Order

To further characterize the influence of acids on the RM wall, we evaluated the order parameter (*S*_CS_), which quantifies the alignment of SDS hydrocarbon tails relative to the local radial direction of the RM (Figure 5a). Higher values of *S*_CS_ indicate more coherent tail alignment. As reported in Figures 5b and S9a, the addition of acids consistently reduces *S*_CS_, implying that acids promote a more disordered packing of the RM wall, with HAuCl_4_ exerting the strongest perturbation.

**Figure 5:**
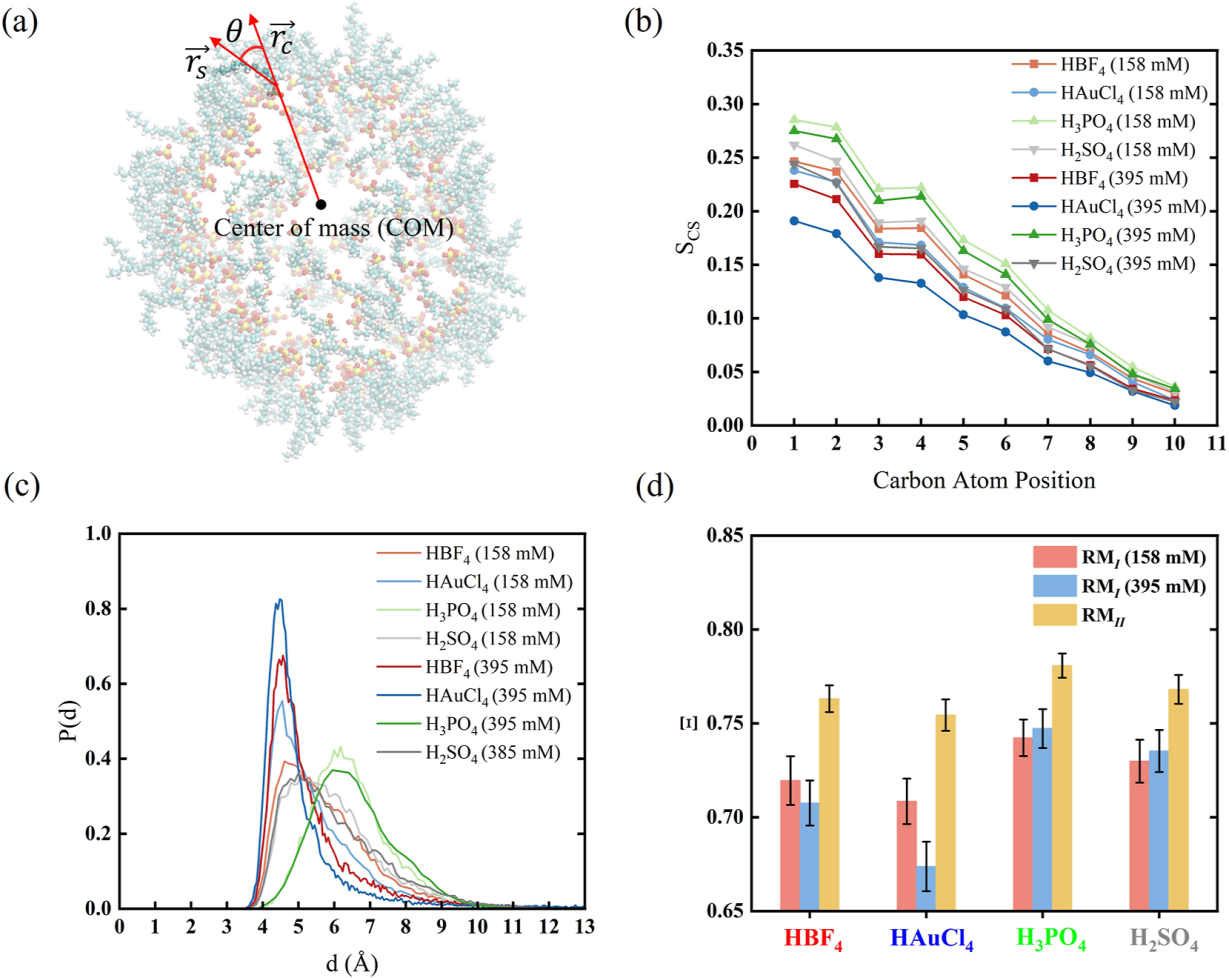
(a) The schematic diagram of the calculations for order parameters (*S*_CS_) in RMs. The θ is the angle between the vector $\vec{r_{c}}$ and $\vec{r_{s}}$ . The $\vec{r_{c}}$ represents the vector between the sulfur atom of a SDS surfactant and the center of mass (COM) of all SDS surfactants, and $\vec{r_{s}}$ represents the vector between the selected carbon atom of a SDS surfactant and the center of mass (COM) of all SDS surfactants. (b) represents *S*_CS_ for RM_I_. (c) Probability density distribution of the nearest distance (*P*(*d*)) between each two sulfur atoms of headgroups for RM_I_. (d) Convexity (Ξ) of RM_I_ and RM_II_. The acid concentrations used in RM_I_ are 158 mM and 395 mM, respectively. The acid concentration used in RM_II_ is 193 mM.

To complement this analysis, we examined the probability density distribution of the nearest sulfur–sulfur distances (*P*(*d*)), which reflects the spatial organization of the SDS headgroups. Figures 5c and S9b show that both HBF_4_ and HAuCl_4_ decrease the average S–S separation, indicating a less homogeneous distribution of headgroups at the interface. We also quantified the convexity of the RMs, Ξ, defined as the ratio of the RM volume to that of its convex hull:

11 $$\Xi =\frac{V\left(S\right)}{V\left(CH\left(S\right)\right)}$$

where *S* denotes the micellar surface. Ξ measures the extent of interfacial indentations and corrugations and therefore provides a geometric proxy for surfactant packing. As illustrated in Figure 5d, Ξ follows the same qualitative trend observed for *S*_CS_ and *P*(*d*). Heterogeneous packing of headgroups and tails increases the interfacial void space, lowering Ξ. Consequently, RMs containing HBF_4_ and HAuCl_4_ exhibit smaller Ξ values than those loaded with H_3_PO_4_ and H_2_SO_4_.

Increasing the acid concentration in the RMs further diminishes *S*_CS_, reflecting enhanced tail misalignment. In the case of HAuCl_4_, the strong accumulation of the AuCl_4_^–^ anion at the water–toluene boundary compresses the interfacial headgroup region, reducing both the S–S distances and Ξ.

By contrast, higher concentrations of H_3_PO_4_ and H_2_SO_4_ yield slightly more homogeneous headgroup distributions, likely due to increased incorporation of H_3_PO_4_ and HSO_4_^–^ species between adjacent headgroups.

#### Acids Modulate Interfacial Tension

To investigate how acids influence interfacial properties in the limit of large RMs, we employed bilayer models BI_I_ with interfacial acid concentrations matched to those in RM_I_ (Table S3). Bilayers offer a practical advantage in enabling a more direct evaluation of interfacial tension (IFT). The behavior of the tail-order parameter in the bilayers, *S*_Z_, mirrors that of *S*_SC_ in the micelles (Figure S9c,d), and the SDS headgroup distributions are likewise consistent (Figure S9b). This correspondence confirms that the acids exert comparable interfacial effects on both geometries.

At fixed surfactant concentration, the impact of the acids on the IFT depends sensitively on both their concentration and molecular characteristics including polarizability. For HBF_4_ and H_2_SO_4_, the IFT increases with acid concentration before reaching a plateau (Figure 6a). In contrast, HAuCl_4_ causes a slight initial increase followed by a continuous decrease at concentrations above 100 mM. H_3_PO_4_, being neutral at the interface and therefore ineffective at modifying electrostatic screening, yields IFT values close to those of the acid-free system across the entire concentration range. Overall, the IFT values γ follow

$$\gamma \left(HBF_{4}\right)>\gamma \left(H_{2}SO_{4}\right)>\gamma \left(HAuCl_{4}\right)>\gamma \left(H_{3}PO_{4}\right)$$

**Figure 6:**
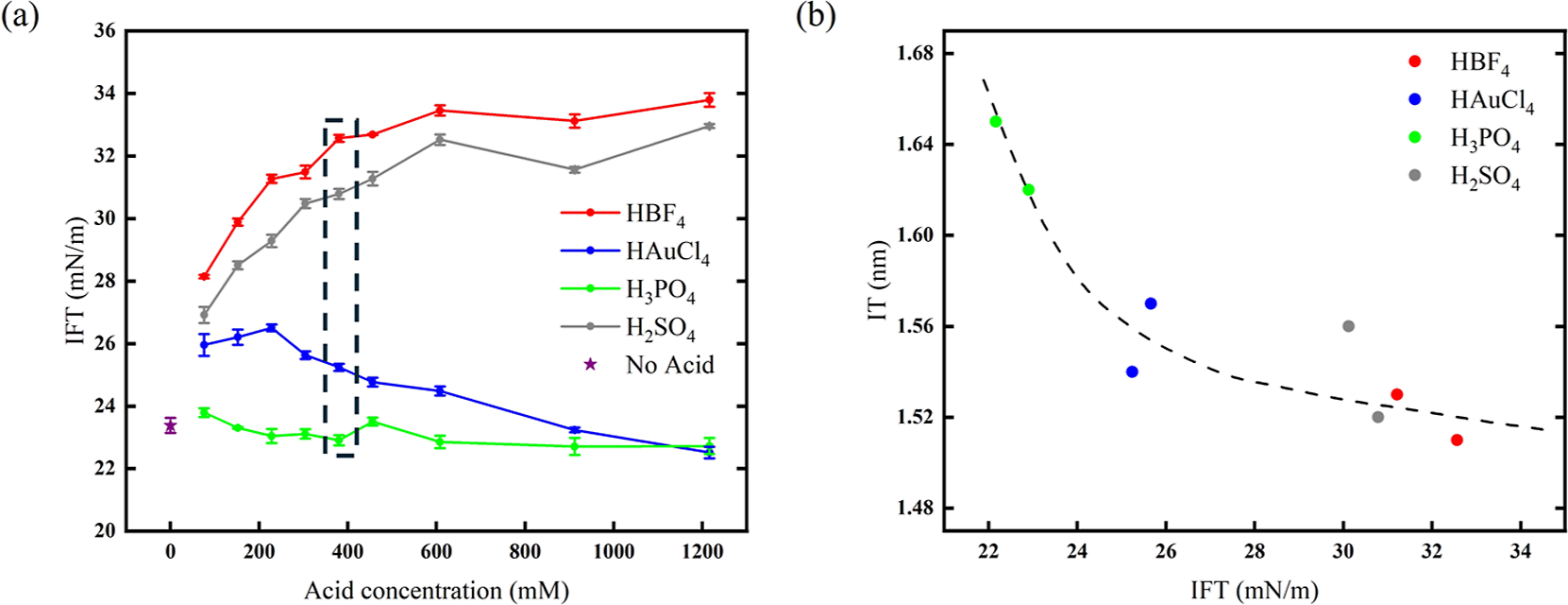
(a) The interfacial tension (IFT) for BI_I_ with different concentrations. Dashed black boxes refer to the BI_I_ at a specific concentration imitating the interfacial environment of RM_I_, where acid concentration is 380 mM. (b) The inverse correlation between the IFT and interfacial thickness (IT) in SDS bilayers, where acid concentrations are 380 mM in BI_I_ and 360 mM in BI_II_, respectively.

The observed IFT trend can be rationalized in terms of the molecular properties of the corresponding species. The strong interfacial exclusion of BF_4_^–^ and HSO_4_^–^ reflects their pronounced hydrophilicity and strong hydration, which favors their retention within the aqueous core and results in relatively high IFT values. In contrast, AuCl_4_^–^ is a larger, more polarizable anion with softer charge distribution, which promotes partial adsorption at the water–toluene interface and thereby lowers the interfacial tension. However, electrostatic repulsion between AuCl_4_^–^ and the negatively charged surfactant headgroups limits this adsorption to low AuCl_4_^–^ concentrations, reducing its impact on the IFT. H_3_PO_4_ is predominantly present as a neutral molecular species and therefore exhibits minimal interfacial activity, leading to IFT values comparable to those observed in the absence of acid.

As previously reported, the magnitude of the IFT is a critical parameter governing the stability, size, and reaction kinetics of the micellar systems. Low IFT results in a highly fluid and deformable surfactant interface, which facilitates rapid intermicellar exchange and enhances mass transfer.^103^ This behavior is particularly advantageous in applications such as fast-kinetic nanoreactors and enhanced oil recovery, where efficient molecular diffusion and dynamic interface adaptability are essential. In contrast, high IFT is associated with increased interfacial rigidity, leading to more stable and less deformable structures. This property can be exploited to create well-defined structural barriers or to template complex morphologies.^104^ Such characteristics are strategically utilized in sustained-release drug delivery systems and nanomaterial templating, where maintaining structural integrity and preventing the premature leakage of encapsulated species are crucial.

#### Connecting Interfacial Tension and Electrostatic Properties

The IFT (γ) is closely related to electrostatic potential difference (Δψ), which we have previously discussed. Higher values of the potential difference are associated with a reduced γ, in agreement with the Lippmann equation,^105^ which describes how the IFT between two immiscible liquids (such as oil and an aqueous electrolyte) changes with the applied potential difference across the interface. Larger values of the potential difference ΔΨ are also associated with ion accumulation at the micellar wall, which increases the interfacial thickness (IT). Interfacial tension and interfacial thickness IT are therefore also inversely correlated (Figure 6b). HBF_4_ and H_2_SO_4_, which most strongly increase the IFT, also yield the thinnest interfacial regions. Conversely, HAuCl_4_ and H_3_PO_4_ induce only minor reductions in interfacial thickness Figure S11. The hierarchy of interfacial thickness for the bilayers

$$IT\left(HBF_{4}\right)<IT\left(H_{2}SO_{4}\right)<IT\left(HAuCl_{4}\right)<IT\left(H_{3}PO_{4}\right)$$

closely matches that identified in the RMs (Figure S4), indicating that trends in the bilayers provide reliable guidance on the corresponding behavior in micelles.

As highlighted in previous studies,^106–108^ acids modify the IFT through changes in interfacial hydrogen bonding and ion-bridging interactions (Figure S12), elaborated further in the Supporting Information. To explore how the IFT depends on surfactant loading, we computed the IFT as a function of SDS concentration at fixed acid concentration (Figure S13a), including a comparison with NaCl as a benchmark electrolyte. Our interfacial tension for the water–toluene boundary without surfactant agrees with literature values.^109^

Across all SDS concentrations, NaCl causes a mild increase in IFT, consistent with the repulsion of Cl^–^ from the interface. Remarkably, only HAuCl_4_ reduces the IFT at low SDS concentrations (Figure S13a), owing to the substantial interfacial adsorption of AuCl_4_^–^ driven by its large polarizability consistent with the principle that highly polarizable ions lower the IFT.^110^ By contrast, BF_4_^–^ exhibits negligible interfacial adsorption (Figure S13d) and remains largely in the aqueous phase due to its hydrophilicity and repulsion from the negatively charged SDS headgroups. HSO_4_^–^ similarly avoids the interface, though it readily forms stable (HSO_4_^–^)_*n*_ clusters in both bilayers and micelles (Figures S3d,h and S13f). H_3_PO_4_ adsorbs appreciably only in the presence of surfactants (Figure S13g), facilitated by hydrogen bonding between H_3_PO_4_ and the headgroups.

Therefore, as the surfactant concentration increases, the interfacial tension is reduced, with higher values for the IFT upon addition of NaCl, HBF_4_, and H_2_SO_4_. In the case of HAuCl_4_, a different behavior is found: below a certain SDS concentration, the addition of HAuCl_4_ reduces the IFT, while above it increases the IFT in line with the other acids or the salt.

#### Possible Consequences for the Design of RMs for Environmental Remediation and Sustainable Nanomaterials

Previous studies have shown that the addition of salts or acids can be exploited to design RMs capable of sequestering contaminants from natural water sources. For instance, Pandit and Basu^111^ reported that salt addition modulates removal efficiency and reduces micellar size via “dewatering” and interfacial charge screening, thereby affecting dye encapsulation. Similarly, Eastoe et al.^112^ demonstrated that the introduction of hydrochloric acid (HCl) into reverse micellar systems in green solvents, such as compressed propane, enables the formation of stable, diamond-shaped copper nanoparticles. Here, we show that the effect of acid addition is strongly dependent on the nature of the counterions. In all cases, protons preferentially localize at the micellar interface, screening the negative charge of the SDS headgroups and promoting stabilization. However, the extent to which acid anions partition to the interface governs the resulting interfacial properties. Specifically, acids with greater interfacial affinity can either increase or decrease the surface tension and modify the interfacial thickness. When anions accumulate more strongly at the interface, a reduction in interfacial tension is observed, accompanied by an increase in interfacial thickness and the formation of more rigid, stable micellar shells. This, in turn, favors the formation of larger and more stable micelles. Further work is required to quantitatively elucidate the relationship between acid identity and RM size.

Overall, we expect that the trends observed here are likely transferable to other RM systems formed by anionic surfactants with similar headgroup chemistry and oil phases of comparable polarity to toluene. For example, AOT reverse micelles in water have also been widely employed as nanoreactors. In such systems, similarly to SDS RMs, a polar water core is separated from the nonpolar oil phase by a shell of anionic surfactant molecules. Acids similar to those examined here could exhibit comparable affinities for the RM interface and could therefore modulate interfacial properties of the assemblies, including interfacial tension as well as micellar size and stability. A systematic investigation of such alternative anionic surfactant–solvent systems will be the subject of future work.

## Conclusions

In this work, we have systematically elucidated how acid identity, dissociation behavior, and concentration govern the structure, electrostatics, and dynamics of SDS-based reverse micelles and their large-size bilayer analogues. By combining micellar models of different sizes as well as bilayer systems that allow direct access to interfacial tension, we establish a unified molecular picture linking ion-specific interfacial partitioning to macroscopic interfacial properties. Across all systems studied, reverse micelles self-assemble robustly, yet their interfacial organization is highly sensitive to the nature of the acid additives. Strong acids that dissociate fully (HBF_4_, HAuCl_4_, and H_2_SO_4_) drive marked proton accumulation at the micellar wall, partially or fully replacing sodium counterions and reshaping the interfacial charge distribution. In contrast, nondissociated H_3_PO_4_ behaves fundamentally differently: its tendency to form oligomeric species limits its interfacial activity, preserves sodium-dominated charge compensation, and produces thicker interfacial regions. These distinct ion-specific distributions translate directly into measurable changes in electrostatic potential, water orientation, and screening efficiency across the micellar and bilayer interfaces. Beyond modifying the local structure, acids also influence interfacial dynamics and flexibility. Increased acid loading consistently reduces SDS headgroup mobility and tail ordering, reflecting the formation of persistent hydrogen-bond networks involving hydrated protons and acid-derived anions. In SDS bilayer models, acids modulate the IFT according to the following trend: γ­(HBF_4_) >γ (H_2_SO_4_) >γ (HAuCl_4_) >γ (H_3_PO_4_). The larger IFT is accompanied by a slight decrease of the interfacial thickness, as well as by a decrease in the interfacial electrostatic potential. Overall, the reduced flexibility of the micellar wall, as well as the decrease in the interfacial tension, may have an impact on the morphology and stability of RMs, eventually leading to larger RMs with increased stability. Altogether, our results underline that acid additives cannot be treated as simple pH modifiers or background electrolytes in confined amphiphilic systems. Instead, their molecular characteristics, including polarizability, hydrogen-bonding propensity, aggregation tendency, and dissociation state, control interfacial structure and mechanics. Our insights provide a molecular basis for tuning reverse micelle and emulsion properties in applications ranging from nanoparticle synthesis and catalysis to separation processes, where subtle changes in interfacial chemistry can dictate macroscopic behavior.

## Supplementary Material



## Notes

Species distribution inside the RM_II_; sensitivity test of additional 10 ns simulations for RM_I_; two-dimensional number density map of acidic anions for RMs; mass density profile of water and toluene for RMs; electrostatic potential of BI_II_ and RM_II_ with water orientation of BI_II_; charge density and accumulated charge for RM_I_; diffusional coefficient for RM_II_; biexponential fitting of hydrogen-bond lifetimes; interfacial arrangement of SDS headgroups and tails for RM_II_ and BIs; equilibration test of IFT in BI_I_; mass density profile of water and toluene for BIs; average of H-bonds and radial density function for SDS headgroups in BI_I_; IFT varied with SDS concentrations; potential of mean force of pulling one acidic species; and comparison of acidic species distributions, interfacial properties, and electrostatic potential between nondissociated, partially dissociated, and fully dissociated H_3_PO_4_ in RMs and BIs (PDF)
