# Supplementary material for: Role of Acids in Stabilizing Reverse Micelles: Insights from Dodecyl Sulfate
Source: Langmuir. 2026 Jun 10;42(24):17247–59. doi: 10.1021/acs.langmuir.6c00158 (PMC13296489; doi:10.1021/acs.langmuir.6c00158)
Supplement: Supplementary file 1 [file la6c00158_si_001.pdf]

Supporting Information for: Role of acids in stabilizing Reverse Micelles: insights from dodecyl Sulfate

Qixuan Li[1] and Marialore Sulpizi*[1]

[1]*Faculty of Physics and Astronomy, Ruhr-University Bochum, Universitätstrasse 150, 44780 Bochum, Germany*

The SDS reverse micelles in the absence of acid.
In the absence of acids, our molecular dynamics simulations show the spontaneous formation of SDS reverse micelles (RMs). However, experimental studies have consistently demonstrated that SDS RMs are difficult to form without the introduction of a third component, such as salts, catalysts, electrolytes, or co-surfactants [1–5]. This apparent discrepancy is possibly due to finite size effects and to the limitations of simplified simulation conditions. The SDS RMs in the absence of acid are not discussed in full details but only considered as limiting case.

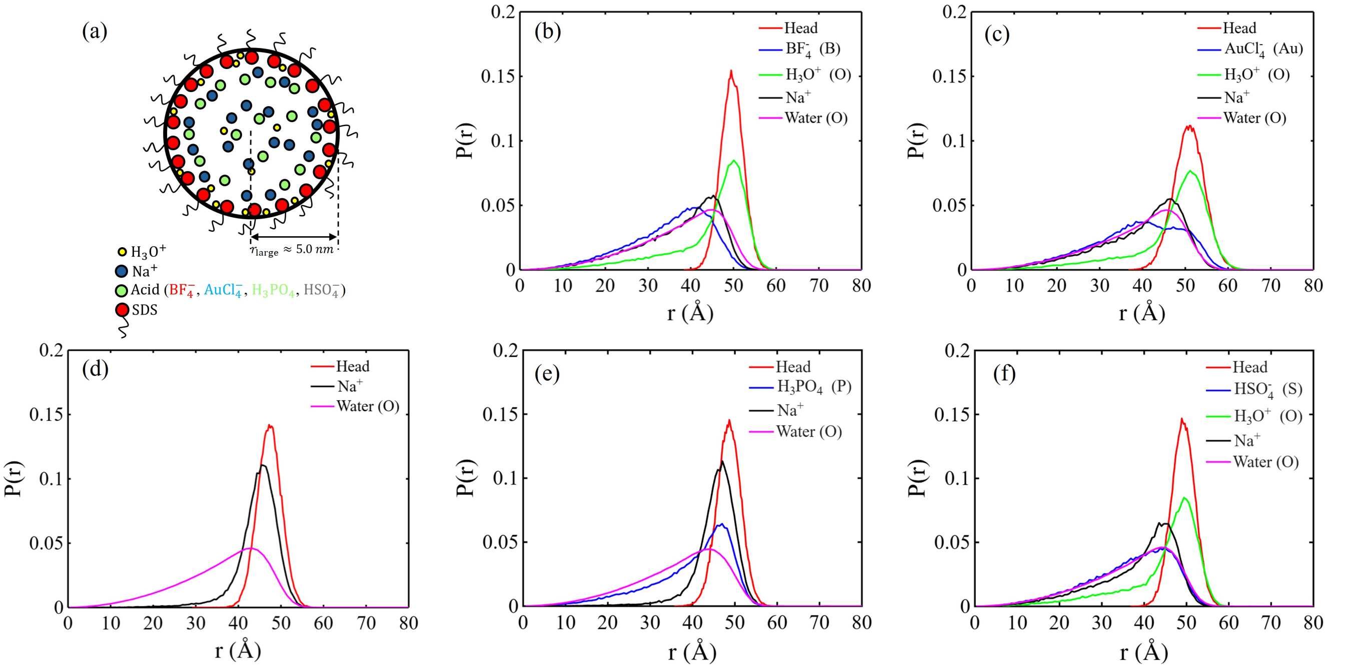

FIG. S1. (a) The schematic acidic species distribution inside the RMs in the case of four acids. (b)-(f) Probability density distribution of the distance ($P(r)$) of the specific types of atoms from the center of mass (COM) for all SDS surfactants. (b), (c), (e) and (f) are the RM_{II} with 193 mM HBF_4, $HAuCl_4$, H_3PO_4 and H_2SO_4 respectively, and (d) represents the RMs without acids.

Ions distributions for the RMs (original 100 ns simulations + additional 10 ns).
In order to confirm that the calculated properties from the simulations reflect equilibrium conditions we have compared the average distributions presented in the main text with the averages from additional 10 ns of simulations. The final structure after 100 ns simulations has been used as initial configuration for additional 10 ns simulation run for each acid case. The $P(r)$ of the additional 10 ns trajectories follows the same distribution as the original results reported in the manuscript, which confirms the equilibration of species distribution inside the RMs after 100 ns equilibration simulations.

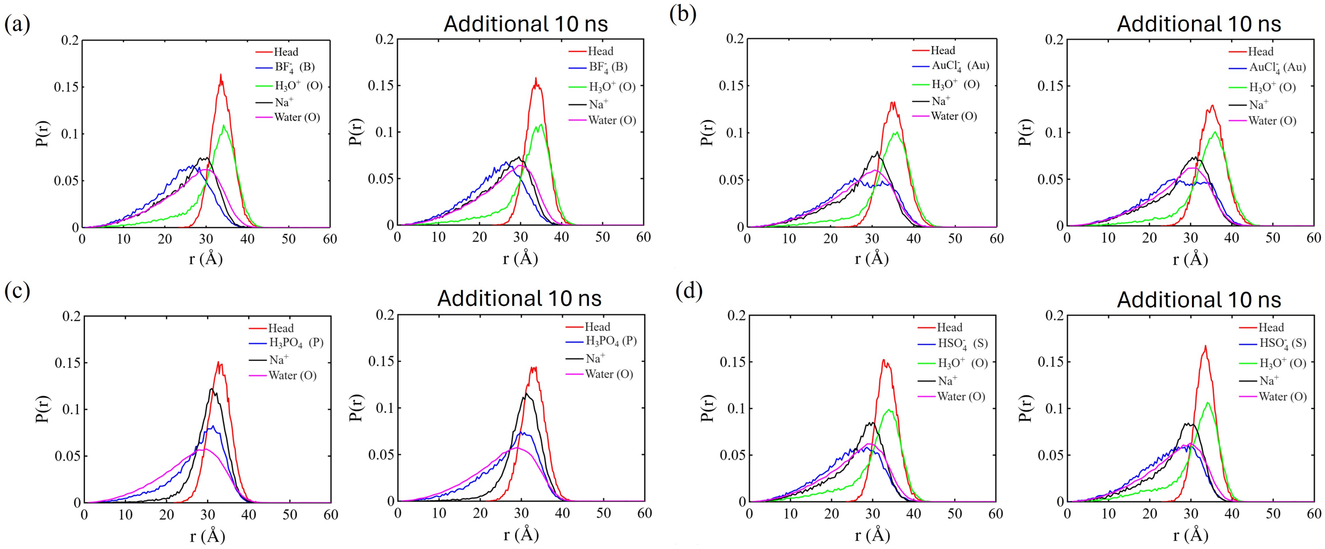

FIG. S2. Probability density distribution ($P(r)$) of the different species as function of the distance from the center of mass (COM) for RM_I. (a) HBF_4, (b) $HAuCl_4$, (c) H_3PO_4 and (d) H_2SO_4, respectively. The panel on the left is the original simulation, while the panel on the right reports the results from the additional 10 ns. In all the cases the acid concentration is 158 mM.

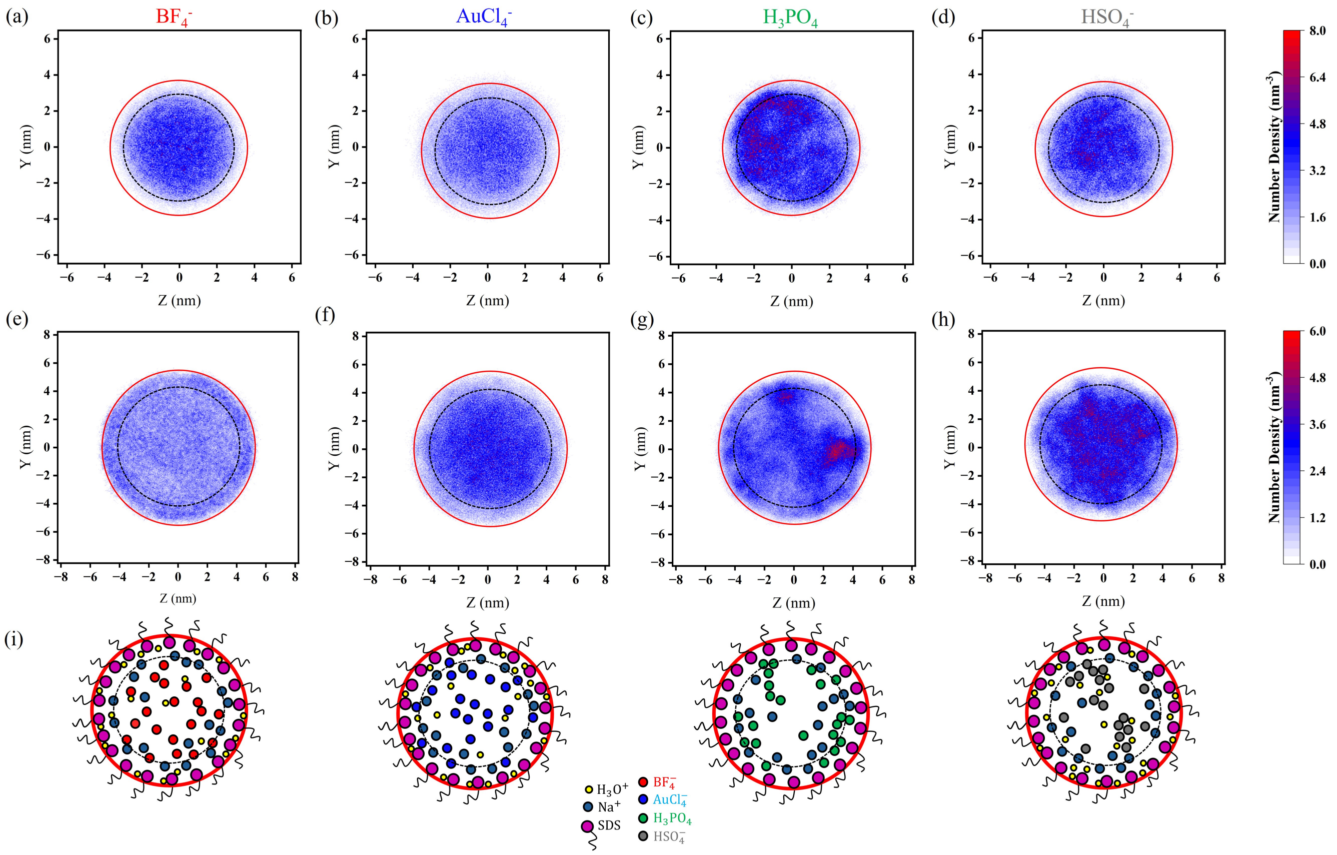

FIG. S3. Two dimensional number density map of acidic anions, BF_4^- (a), $AuCl_4^-$ (b), H_3PO_4 (c) and HSO_4^- (d) in RM_I and BF_4^- (e), $AuCl_4^-$ (f) , H_3PO_4 (g) and HSO_4^- (h) in RM_{II}, respectively. Red solid lines represent the outer boundaries of each acidic anion. Black dashed lines represent the inner region boundaries. The acid concentrations used in RM_I and RM_{II} are 158 mM and 193 mM respectively. (i) The scheme for each species distribution inside the RMs in the case of four acids.

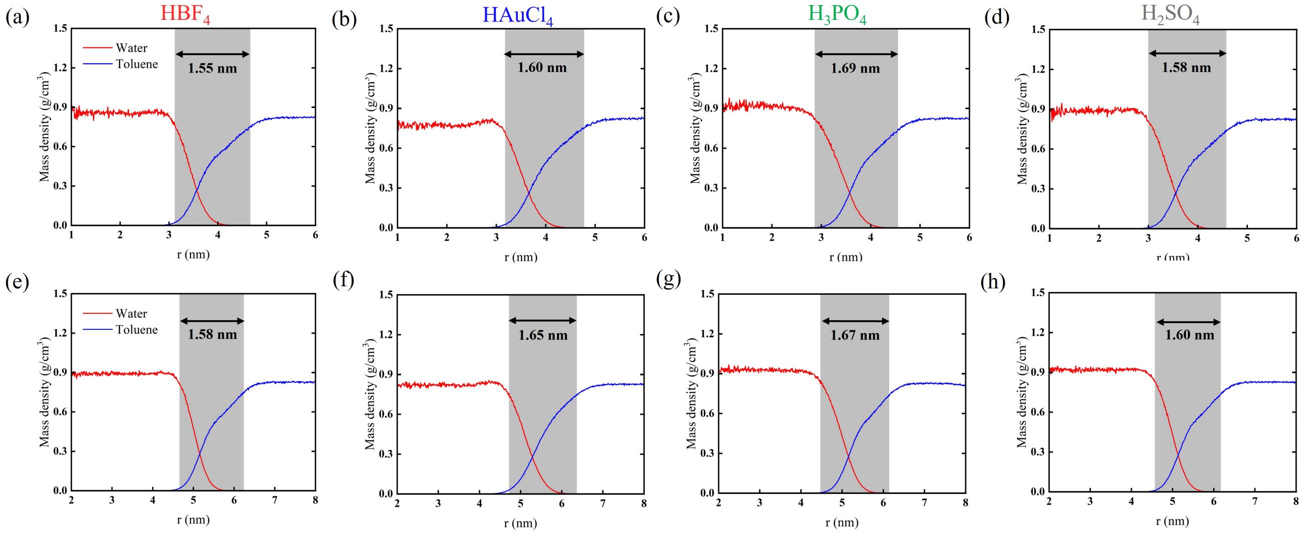

FIG. S4. Mass density profile of water and toluene for RM_I and RM_{II}. The 90-90 interfacial thickness between water and toluene is represented by the gray region for each case: (a) HBF_4 RM_I, (b) $HAuCl_4$ RM_I, (c) H_3PO_4 RM_I, (d) H_2SO_4 RM_I, (e) HBF_4 RM_{II}, (f) $HAuCl_4$ RM_{II}, (g) H_3PO_4 RM_{II}, (h) H_2SO_4 RM_{II}. The acid concentrations used in RM_I and RM_{II} are 158 mM and 193 mM respectively.

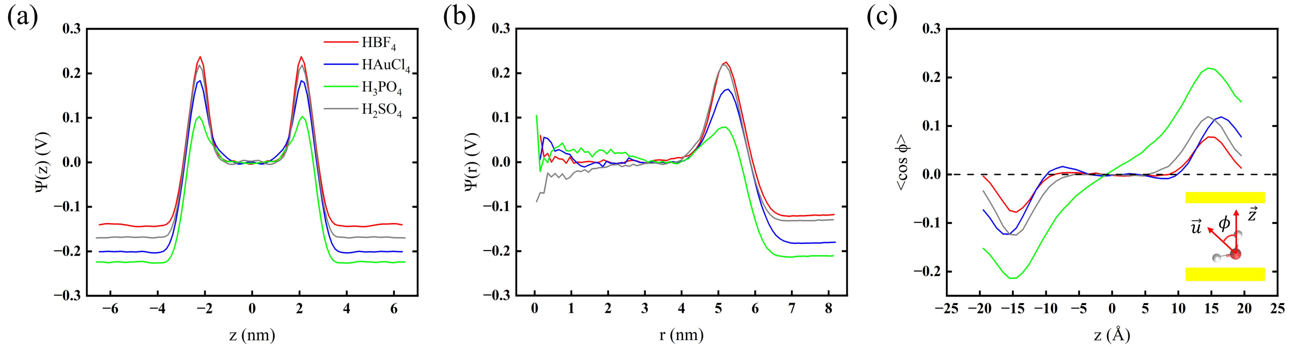

FIG. S5. (a) and (b) Electrostatic potential of BI_{II} ($\psi(z)$) and RM_{II} ($\psi(r)$). (c) Water orientation of BI_{II}. The acid concentrations used in BI_{II} and RM_{II} are 360 mM and 193 mM respectively.

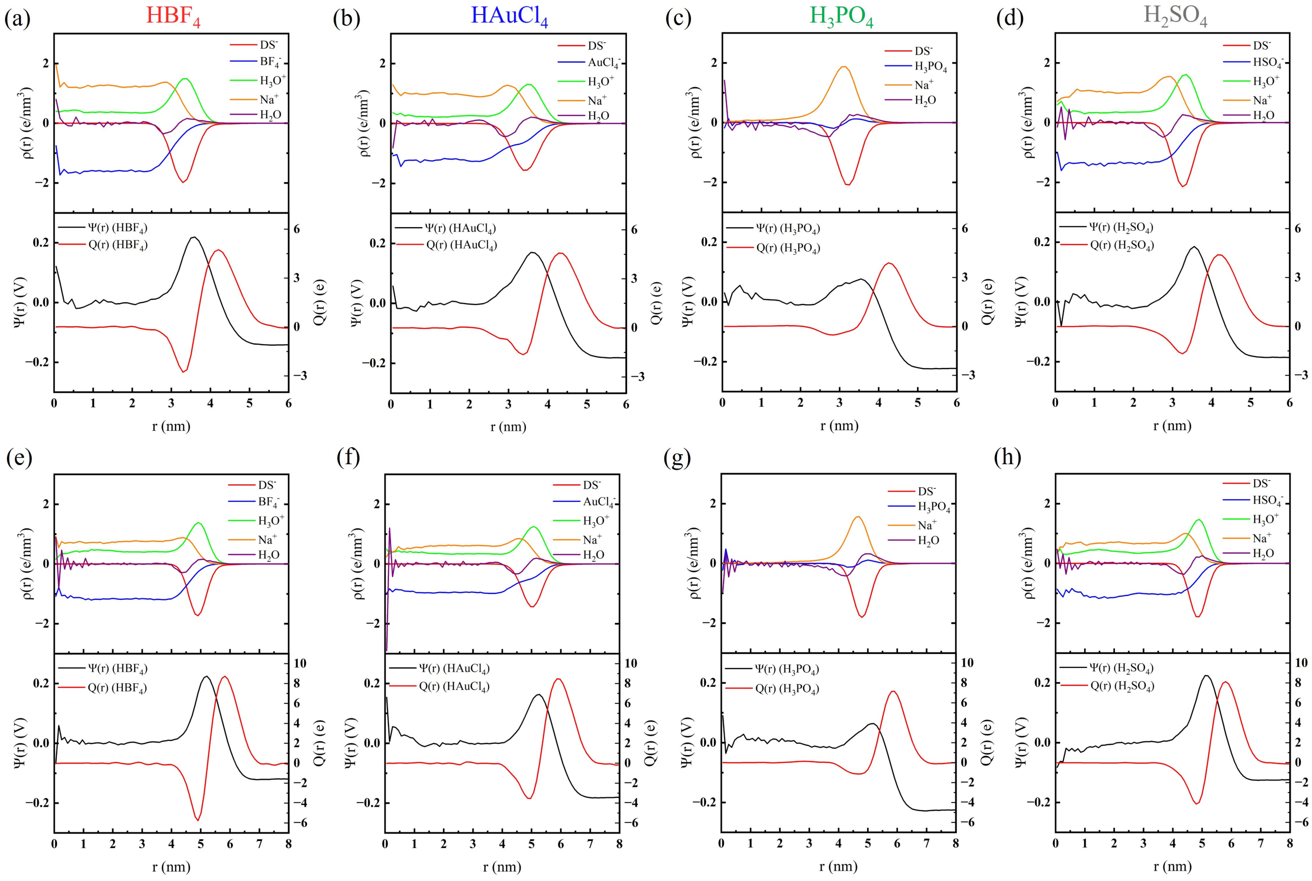

FIG. S6. Each species of charge density ($\rho(r)$) and accumulated charge ($Q(r)$) for RM_I in (a), (b), (c) and (d), for RM_{II} in (e), (f), (g) and (h). The acid concentrations used in RM_I and RM_{II} are 158 mM and 193 mM respectively.

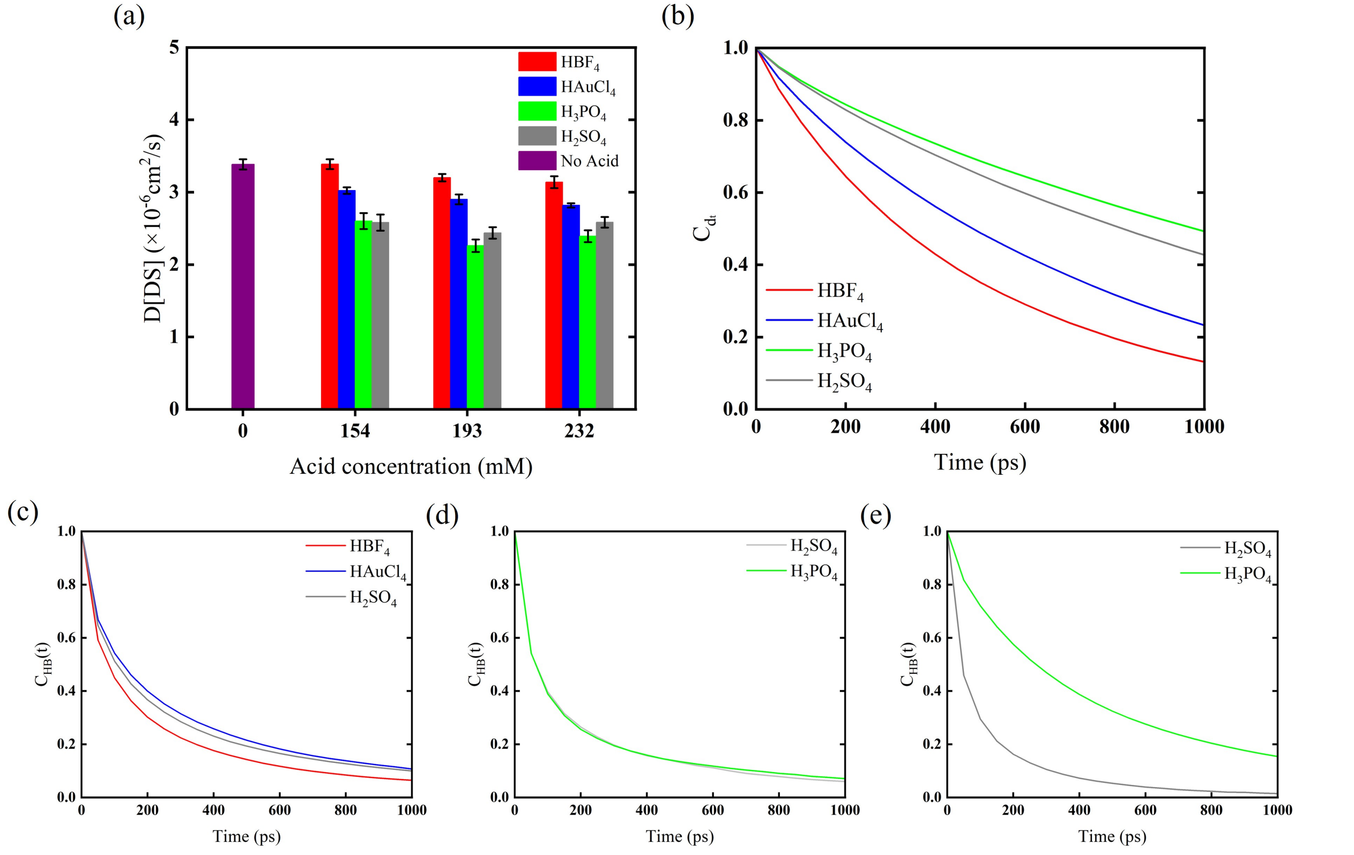

FIG. S7. (a) Diffusional coefficient of SDS surfactants ($D[DS]$) for RM_{II} with different acid concentrations. (b) Autocorrelation function of distance (C_{d_t}), the distance is between selected acidic species and the center of mass (COM) of SDS surfactants. (c-e) Autocorrelation function of hydrogen bonds (H-bonds) ($C_{HB}(t)$), the H-bond is between hydrated protons and head groups of SDS surfactants in (c), the H-bond is between hydrated protons and acidic species (HSO_4^- or H_3PO_4) in (d), the H-bond is between acidic species and acidic species ($HSO_4^- \cdots HSO_4^-$ or $H_3PO_4 \cdots H_3PO_4$) in (e).

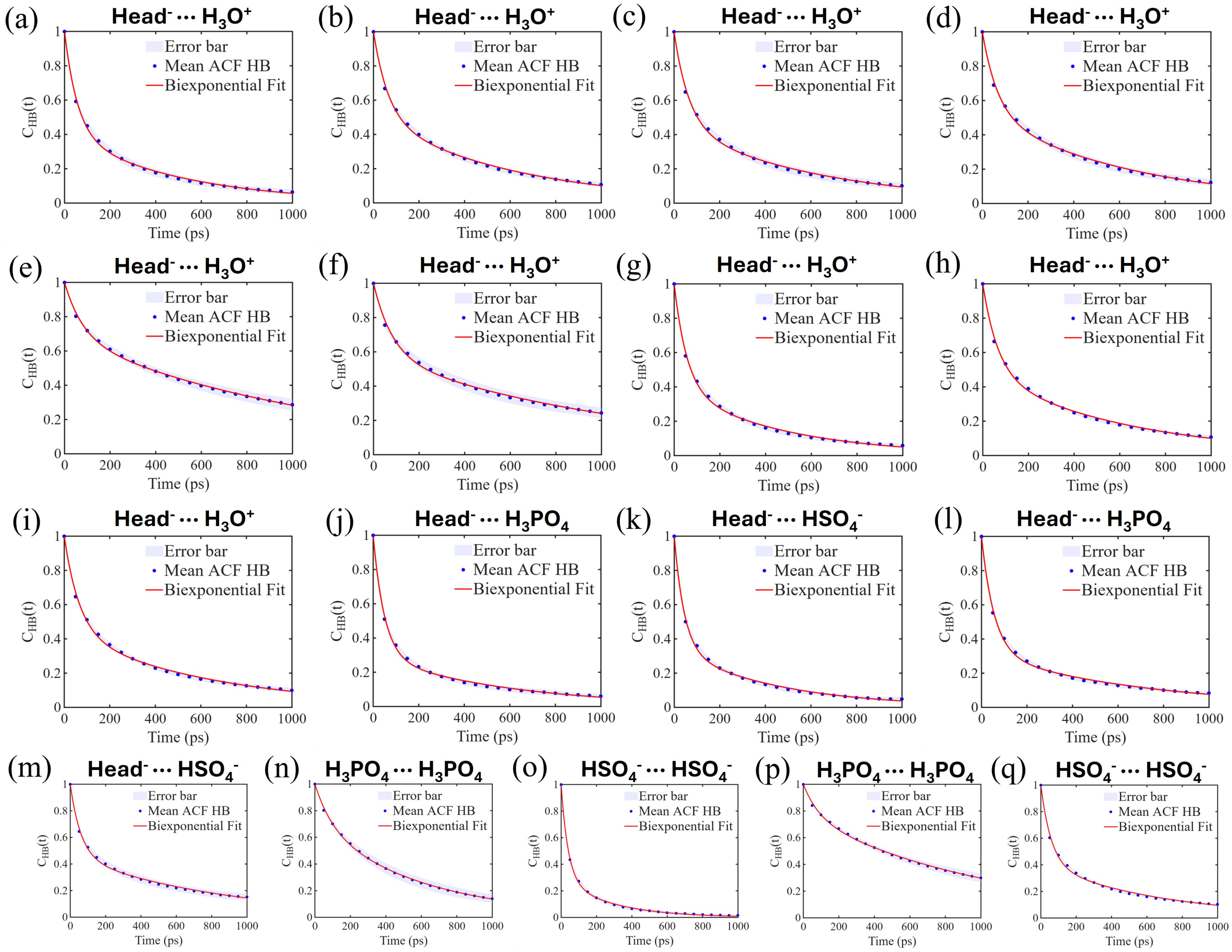

FIG. S8. The biexponential fitting of hydrogen-bond lifetimes in the different cases. (a) $Head^- \cdots H_3O^+$ (RM_I with 158 mM HBF_4), (b) $Head^- \cdots H_3O^+$ (RM_I with 158 mM $HAuCl_4$), (c) $Head^- \cdots H_3O^+$ (RM_I with 158 mM H_2SO_4), (d) $Head^- \cdots H_3O^+$ (RM_I with 395 mM HBF_4), (e) $Head^- \cdots H_3O^+$ (RM_I with 395 mM $HAuCl_4$), (f) $Head^- \cdots H_3O^+$ (RM_I with 395 mM H_2SO_4), (g) $Head^- \cdots H_3O^+$ (RM_{II} with 193 mM HBF_4), (h) $Head^- \cdots H_3O^+$ (RM_{II} with 193 mM $HAuCl_4$), (i) $Head^- \cdots H_3O^+$ (RM_{II} with 193 mM H_2SO_4), (j) $Head^- \cdots H_3PO_4$ (RM_I with 158 mM H_3PO_4), (k) $Head^- \cdots HSO_4^-$ (RM_I with 158 mM H_2SO_4), (l) $Head^- \cdots H_3PO_4$ (RM_I with 395 mM H_3PO_4), (m) $Head^- \cdots HSO_4^-$ (RM_I with 395 mM H_2SO_4), (n) $H_3PO_4 \cdots H_3PO_4$ (RM_I with 158 mM H_3PO_4), (o) $HSO_4^- \cdots HSO_4^-$ (RM_I with 158 mM H_2SO_4), (p) $H_3PO_4 \cdots H_3PO_4$ (RM_I with 395 mM H_3PO_4), (q) $HSO_4^- \cdots HSO_4^-$ (RM_I with 395 mM H_2SO_4). The gray shadows represent the error bars of the mean value of hydrogen-bond autocorrelation function ($C_{HB}(t)$), and red lines are the fitting curves.

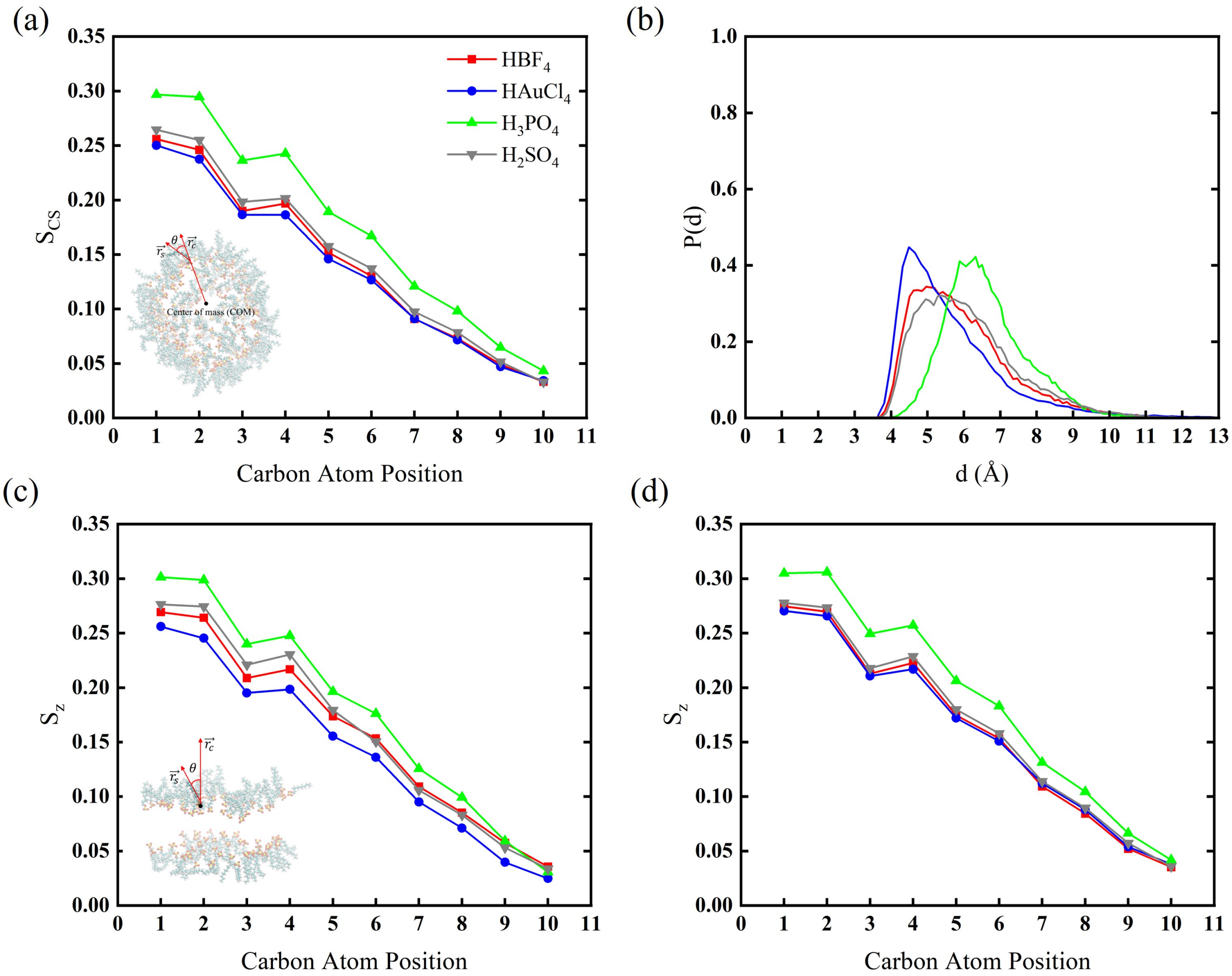

FIG. S9. (a) represents S_{CS} for RM$_{II}$. (b) Probability density distribution of the nearest distance ($P(d)$) between each two sulfur atoms of head groups for RM$_{II}$. The acid concentration used in RM$_{II}$ is 193 mM. (c) The schematic diagram of the calculations for order parameters (S_Z) in SDS bilayers. The θ is the angle between the vector $\vec{r_c}$ and $\vec{r_s}$. The $\vec{r_c}$ represents the vector between sulfur atom of a SDS surfactant and the center of mass (COM) of all SDS surfactants, and $\vec{r_s}$ represents the vector between selected carbon atom of a SDS surfactant and the center of mass (COM) of all SDS surfactants. (c) and (d) represent S_Z for BI$_I$ and BI$_{II}$ respectively. The acid concentrations used in BI$_I$ and BI$_{II}$ are 380 mM and 360 mM respectively.

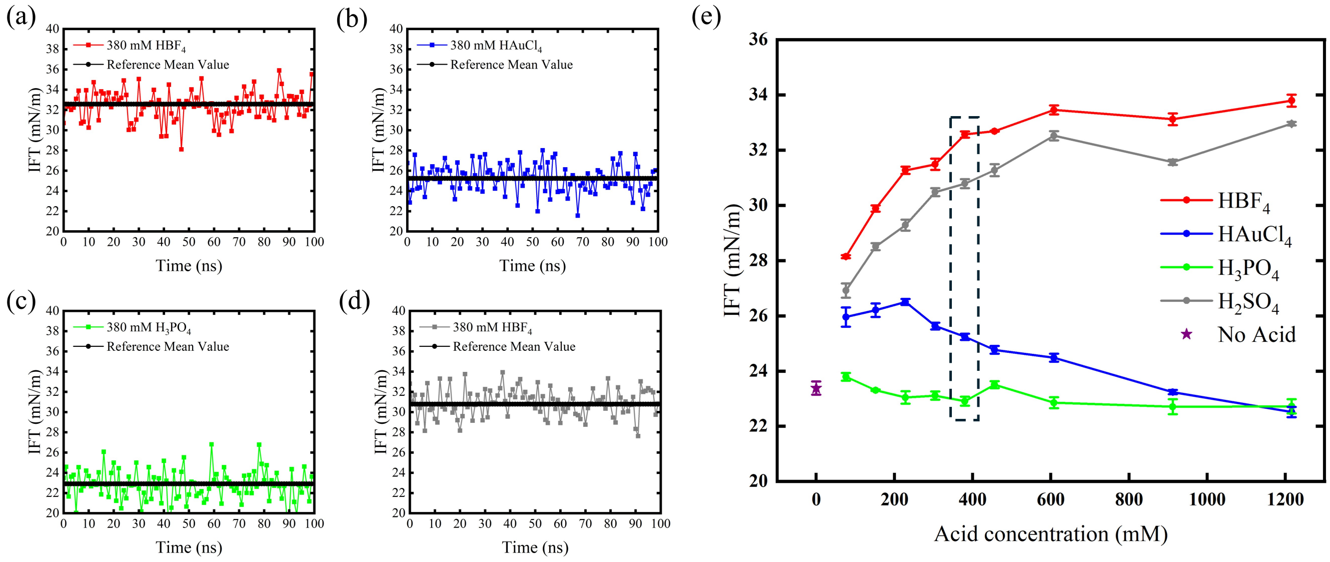

FIG. S10. The equilibration of interfacial tension (IFT) during 100 ns simulations in BI_I. (a) HBF_4, (b) $HAuCl_4$, (c) H_3PO_4 and (d) H_2SO_4. (e) The reference mean values of IFT in the different acid cases refer to the dashed black boxes of BI_I at 380 mM acid concentration.

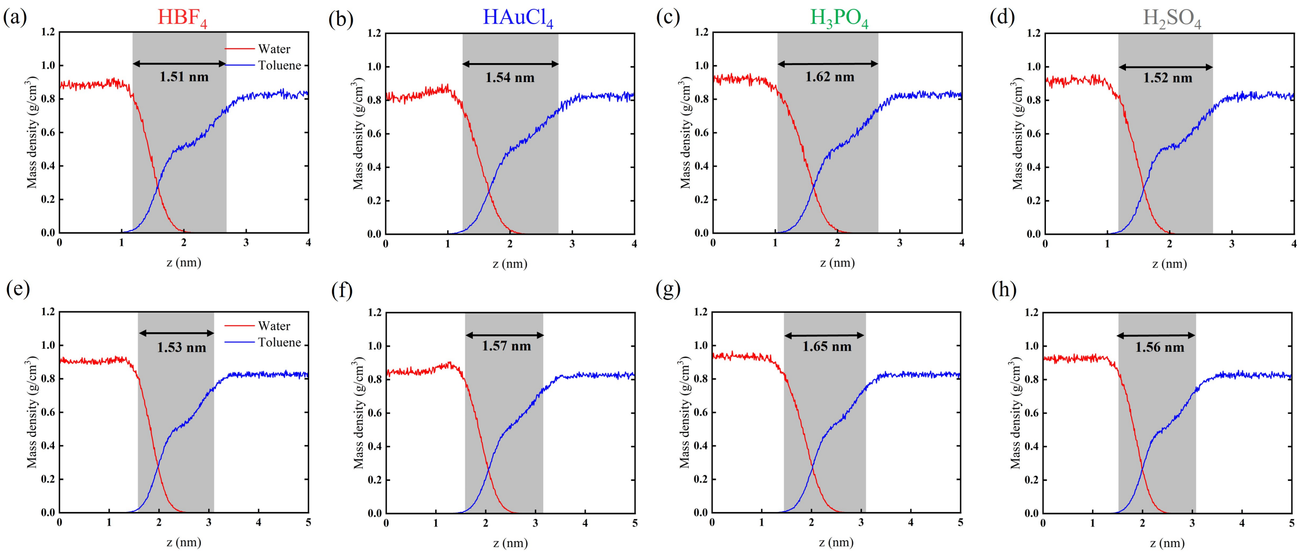

FIG. S11. Mass density profile of water and toluene for BI_I and BI_{II}. The 90-90 interfacial thickness between water and toluene is represented by the gray region for each case: (a) HBF_4 BI_I, (b) $HAuCl_4$ BI_I, (c) H_3PO_4 BI_I, (d) H_2SO_4 BI_I, (e) HBF_4 BI_{II}, (f) $HAuCl_4$ BI_{II}, (g) H_3PO_4 BI_{II}, (h) H_2SO_4 BI_{II}. The acid concentrations used in BI_I and BI_{II} 380 mM and 360 mM respectively.

H-bonds and ion bridges modulate the interfacial tension.

Interfacial tension (IFT) is governed by a balance between hydrogen bonding and electrostatic interactions at the interface. Previous studies have shown that a reduction in interfacial hydrogen bonds leads to an increase in IFT [6]. In contrast, enhanced packing of surfactant headgroups arising from electrostatic screening and ion-bridging effects can decrease the IFT [7]. As shown in Fig. S12(a), the number of interfacial H-bonds is reduced in the presence of HBF_4, $HAuCl_4$ and H_2SO_4. This reduction constitutes a positive contribution to increasing the IFT.

However, increasing acid concentration also leads to a more compact arrangement of SDS headgroups in the cases of HBF_4 and $HAuCl_4$. In particular, $HAuCl_4$ increases the sulfate headgroups density and enhances ion-bridge formation with hydrated protons, as evidenced by the increased sulfur–sulfur radial distribution function (Fig. S12(b)). This enhanced headgroup packing acts as a competing mechanism that lowers the IFT. Moreover, owing to its high polarizability, $AuCl_4^-$ is more likely absorbed at the water-toluene interface, further contributing to a reduction in IFT. Thus, at high $HAuCl_4$ concentration, we found that the IFT decreases slightly and reaches a plateau, reflecting the dominance of electrostatic screening and ion-bridging effects over hydrogen-bond disruption. At lower $HAuCl_4$ concentrations, the reduction in hydrogen bonding remains the dominant factor, leading to a gradual increase in IFT. Consistent with previous reports [8], counterion screening of charged surfactant headgroups promotes interfacial densification, explaining the narrower headgroup distribution and enhanced sulfur–sulfur correlations observed in the $HAuCl_4$ system. By contrast, increasing H_3PO_4 concentration does not significantly alter the number of interfacial hydrogen bonds. As a neutral species, H_3PO_4 does not contribute to electrostatic screening at the interface, resulting in an IFT that remains close to the no-acid case.

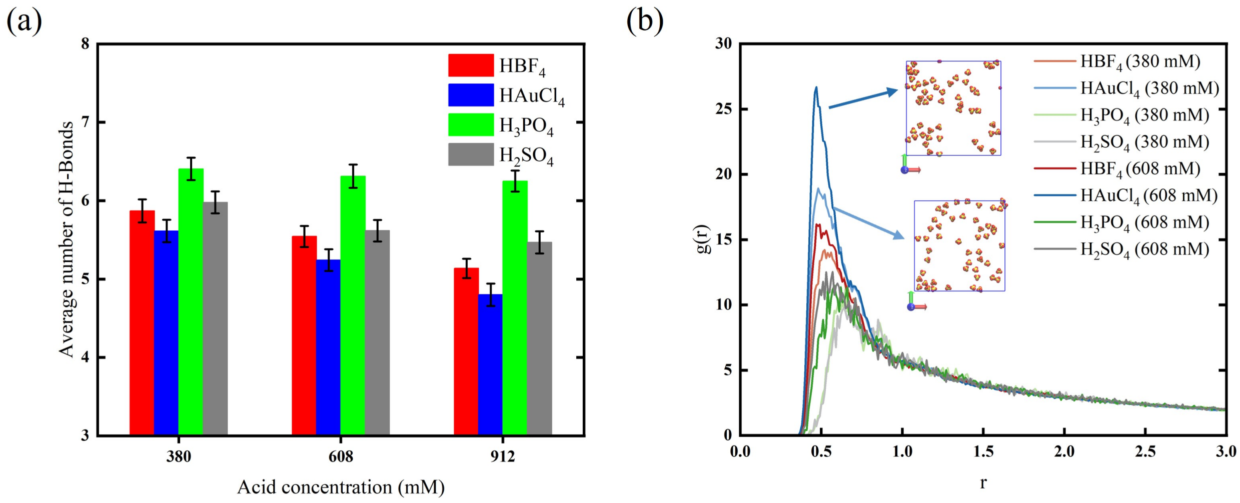

FIG. S12. (a) The average number of interfacial hydrogen bonds (H-bonds) per surfactant in BI_I with different acid concentration. (b) represents the radial density function (RDF) of sulfur and sulfur atoms in the head groups. The acid concentrations used in BI_I is 380 mM and 608 mM respectively.

Effects of acids and salts on interfacial tension at varying SDS concentrations. In the absence of SDS, the addition of HBF_4, H_3PO_4, H_2SO_4 and NaCl slightly increases the IFT at the water–toluene interface (Fig. S13(b)), reflecting their distinct hydrophilicities and diffusivities in the aqueous phase. In contrast, $HAuCl_4$ decreases the IFT even without SDS, as its highly polarizable $AuCl_4^-$ anion preferentially adsorbs at the interface, effectively behaving as a surfactant (Fig. S13(b)).

Upon increasing the SDS concentration, electrostatic repulsion between the negatively charged SDS headgroups and $AuCl_4^-$ ions suppresses $AuCl_4^-$ absorption at water-toluene and weaken the effect of decreasing the IFT. Thus, this is the reason why at an SDS concentration of 304 mM , the IFT in the $HAuCl_4$ system becomes slightly higher than that of the no-acid case. For NaCl, HBF_4, H_2SO_4 and $HAuCl_4$, the accumulation of negatively charged SDS headgroups at the interface promotes the adsorption of cations (Na^+ and H_3O^+) while driving the corresponding anions (BF_4^-, $AuCl_4^-$ and HSO_4^-) toward the aqueous phase.

In the case of H_3PO_4, increasing SDS concentration facilitates the association of $(H_3PO_4)_n$ species with the SDS bilayers (Fig. S13(g)). However, since H_3PO_4 is neutral, does not directly modulate electrostatic interactions at the interface, resulting in a comparatively weak influence on the IFT relative to the other acids.

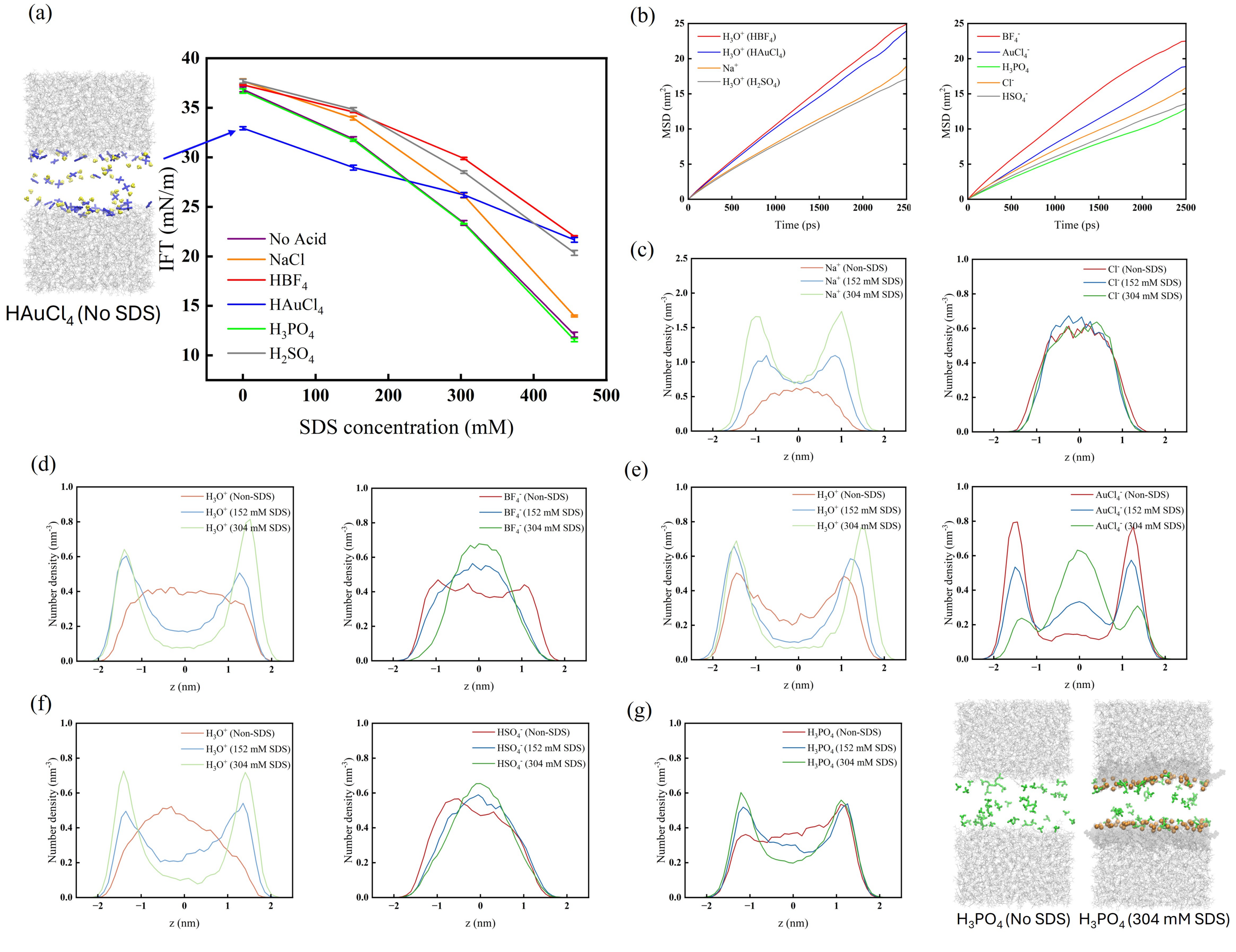

FIG. S13. (a) The interfacial tension (IFT) with fixed acid or salt concentration and varied with SDS concentration. The explicit reduction in the case of $HAuCl_4$ is because the absorption of $HAuCl_4$ at the water-toluene interface (left figure). (b) The average mean square displacement (MSD) for two species for HBF_4, $HAuCl_4$, H_3PO_4, NaCl and H_2SO_4 when no SDS surfactants is present at the water-toluene interface. (c)-(g) represent the number density distribution of acidic species with SDS concentrations of 0, 152 mM and 304 mM, respectively. The fixed acid or salt concentration is 152 mM. The enhanced attraction of H_3PO_4 at the interface can be observed after adding SDS surfactants in (g). The orange beads represent the sulfur atoms in the headgroups and the tails of SDS are shown in transparent black beads. And bright green tubes represent H_3PO_4, blue tubes represent $AuCl_4^-$, yellow beads represent the H_3O^+ and gray lines represent the toluene. Water is not shown for clarity.

Calculation of the potential of mean force for the different acidic species.
The potential of mean force (PMF) was calculated for the different acidic species. From our simulations, the acidic species are consistently observed to reside within the aqueous region. Therefore, to characterize their free-energy profiles, they were pulled from the SDS interface towards the aqueous core, as illustrated in Fig. S14(a).

Because the headgroups of SDS carry the same charge as the acidic anions, strong electrostatic repulsion arises at the interface. Consequently, the anionic species preferentially reside within the aqueous core, where the PMF reaches its lowest value. Nevertheless, an interesting feature emerges for $AuCl_4^-$: a local free-energy minimum is observed near the interface. This indicates a partial preference for interfacial adsorption compared with BF_4^- and HSO_4^-.

In contrast, the PMF profile for H_3PO_4 displays a markedly different behavior. The free energy increases almost linearly as the molecule is moved towards the aqueous core and eventually reaches a higher plateau. This trend suggests that H_3PO_4 exhibits a clear affinity for the interface rather than the internal aqueous region, with an estimated binding free energy of approximately 13.1 kJ/mol.

Further evidence is provided by the probability density distributions of the acidic species (Fig. S14(b)), which confirms that the degree of interfacial adsorption follows the order:

$$H_3PO_4 > HAuCl_4 > H_2SO_4 \approx HBF_4.$$

Overall, negatively charged acidic species tend to avoid the SDS interface due to electrostatic repulsion from the negatively charged headgroups. Notably, $AuCl_4^-$ exhibits a local free-energy minimum at the interface, indicating interfacial adsorption arising from its stronger polarizability and more diffuse charge distribution. By contrast, the PMF profile of H_3PO_4 differs substantially, indicating that this neutral molecule preferentially resides at the SDS interface rather than within the aqueous core. The relatively large binding free energy further supports the stabilization of H_3PO_4 at the interface.

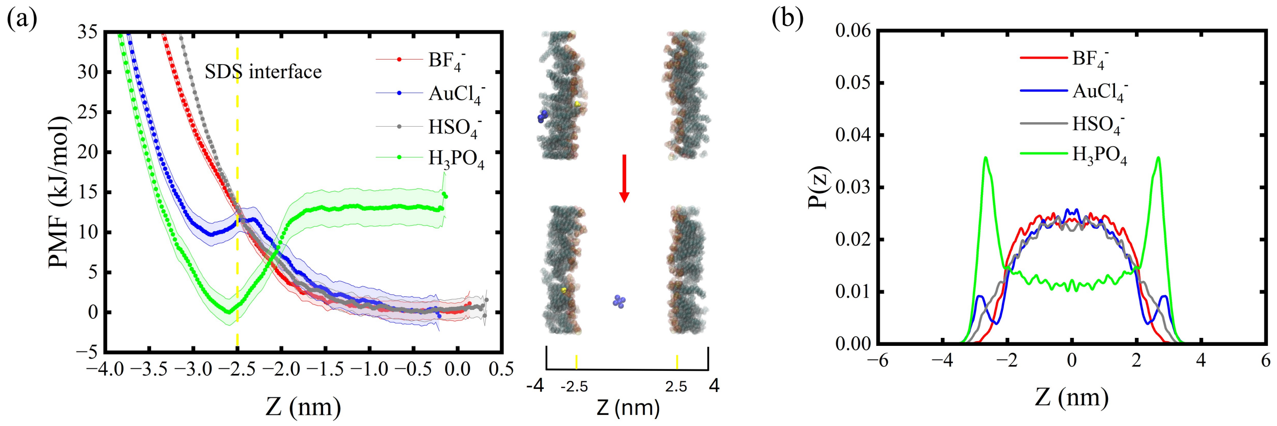

FIG. S14. (a) Potential of mean force (PMF) for pulling one acidic species (BF_4^-, $AuCl_4^-$, HSO_4^- or H_3PO_4) from the SDS interface to the bulk solution (left). The associated error bars (shadows) on the PMF profiles were determined via a bootstrap method [9]. The approximate location of SDS interface is shown as dashed yellow line. The schematic process of pulling one $AuCl_4^-$ from the SDS interface (right). The blue beads represent $AuCl_4^-$ and yellow beads represent the H_3O^+. The acid concentration with one acidic species for each case is 2.6 mM. Water and toluene are not shown for clarity. (b) The probability density distribution ($P(z)$) of acidic species in BIs. Each BI for the calculations of $P(z)$ contains 50 acidic species (130 mM) to make sure the sufficiency of point sampling.

Comparison between non-dissociated, partially and fully dissociated H_3PO_4.
H_3PO_4 is a weak acid, and its degree of dissociation is strongly suppressed at high concentrations. Consequently, only a small fraction of H_3PO_4 can be deprotonated into $H_2PO_4^-$ and H_3O^+. To assess the impact of dissociation, we explicitly compare systems containing non-dissociated, partially and completely dissociated H_3PO_4. As shown in Fig. S15(c) and (d), the dissociation products $H_2PO_4^-$ and H_3O^+ exhibit a stronger tendency to cluster and preferentially adsorb at the micellar interface. Despite this interfacial accumulation, dissociated H_3PO_4 yields SDS diffusivities, spatial distributions, and electrostatic potential profiles within RMs and BIs that are comparable to those observed for non-dissociated H_3PO_4.

Interesting, however, dissociated $(H_3PO_4)_n$ clusters lead to an increase in interfacial tension (IFT), which can be attributed to their aggregation at the interface and the associated enhancement of interfacial roughness. At higher concentrations, $(H_3PO_4)_n$ aggregates increasingly form bridges between opposing SDS bilayers, a configuration that instead reduces the IFT. Overall, the dissociation products $H_2PO_4^-$ and H_3O^+ tend to associate into stable interfacial clusters ((Fig. S16(c)), that can be attracted to the negatively charged SDS headgroups. Nonetheless, this clustering has a limited impact on the micellar interfacial structure and dynamics, with a modest increase in the interfacial tension (Fig. S16(i)).

In addition, we investigated RM_I and BI_I in the case of dissociated H_3PO_4 mixed with non-dissociated H_3PO_4 at a ratio of 1:1, where we defined as partially dissociated H_3PO_4. It is noted that dissociated H_3PO_4 promote the formation of $(H_3PO_4)_n$ clusters associated with non-dissociated H_3PO_4 in the RMs, as shown in Fig. S17(a). And we can observe that non-dissociated H_3PO_4 is closer to the headgroups compared to $H_2PO_4^-$, which may indicate the non-dissociated H_3PO_4 can affect the interfacial properties more dominantly. Moreover, similar to the case of non-dissociated H_3PO_4 and dissociated H_3PO_4 as previous discussed, the RM_I in the case of partially dissociated H_3PO_4 shows the similar trend in micellar flexibility and headgroups distribution (Fig. S17(c) and (d)). Interestingly, we found that the value of IFT in the case of partially dissociated H_3PO_4 is very close to the case of non-dissociated H_3PO_4 at low concentration, which could be explained that non-dissociated H_3PO_4 can affect IFT more significantly because of its stronger affinity to the SDS interface respect to $H_2PO_4^-$, consistent with what we observed in the RMs.

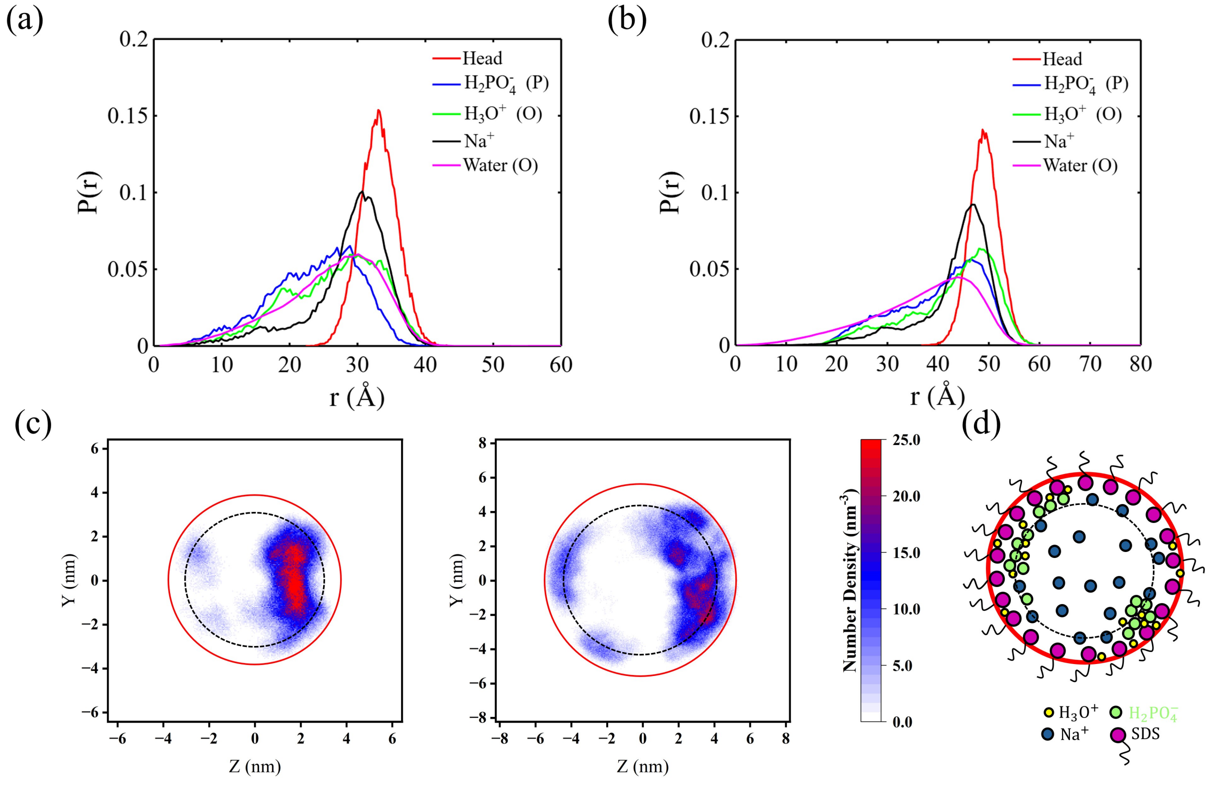

FIG. S15. Probability density distribution of the distance of the specific types of atoms from the COM for all SDS surfactants in the case of dissociated H_3PO_4. (a) RM_I and (b) RM_{II}. (c) Two dimensional number density map of $H_2PO_4^-$, RM_I (left) and RM_{II} (right). (d) The scheme for each species distribution inside the RMs in the case of dissociated H_3PO_4. The H_3PO_4 concentrations used in RM_I and RM_{II} are 158 mM and 193 mM, respectively.

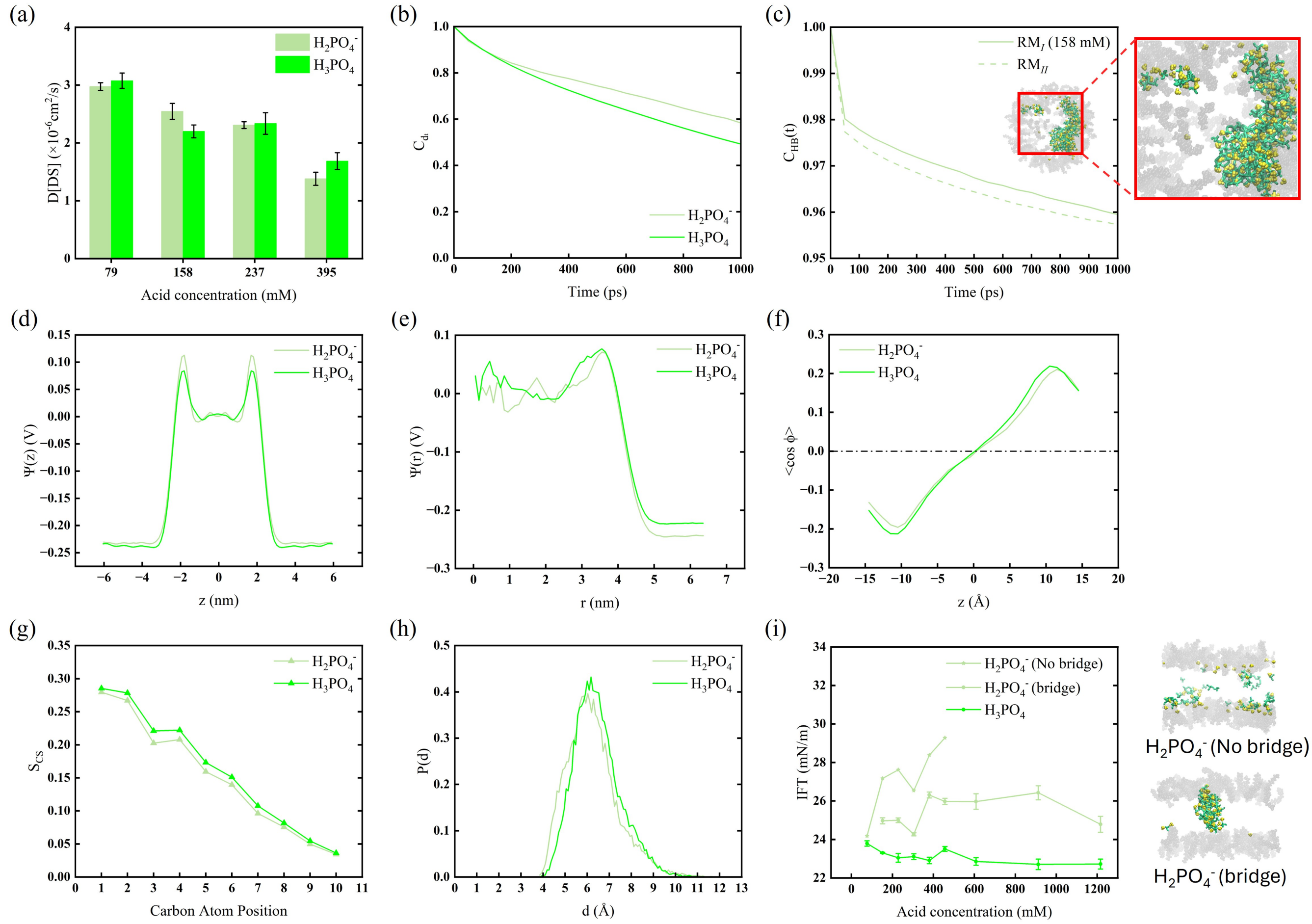

FIG. S16. Comparison of the properties between non-dissociated H_3PO_4 (represented by H_3PO_4) and dissociated H_3PO_4 (represented by $H_2PO_4^-$) in RM_I and BI_I. (a) Diffusional coefficient of SDS surfactants ($D[DS]$) with different concentration. (b) Autocorrelation function of distance (C_{d_t}). (c) Autocorrelation function of hydrogen bonds (H-bonds) ($C_{HB}(t)$), the H-bond is between $H_2PO_4^-$ and H_3O^+. Right snapshot is the last frame of RM_I. (d) and (e) Electrostatic potential of BI_I ($\psi(z)$) and RM_I ($\psi(r)$). (f) Water orientation of BI_I. (g) represents S_{CS} for RM_I. (h) Probability density distribution of the nearest distance ($P(d)$) between each two sulfur atoms of head groups for RM_I. (i) The interfacial tension (IFT) for BI_I with different concentration between non-dissociated H_3PO_4 and dissociated H_3PO_4. The snapshots of bridge and no-bridge effect in the case of of $H_2PO_4^-$ and H_3O^+ are shown in the right. (b)-(h) The H_3PO_4 concentrations used in RM_I and RM_{II} are 158 mM and 193 mM respectively, and in BI_I is 380 mM. And $H_2PO_4^-$ are represented by dark green tubes, H_3O^+ are represented by yellow beads and SDS surfactants are shown in transparent black beads. Toluene and water are not shown for clarity.

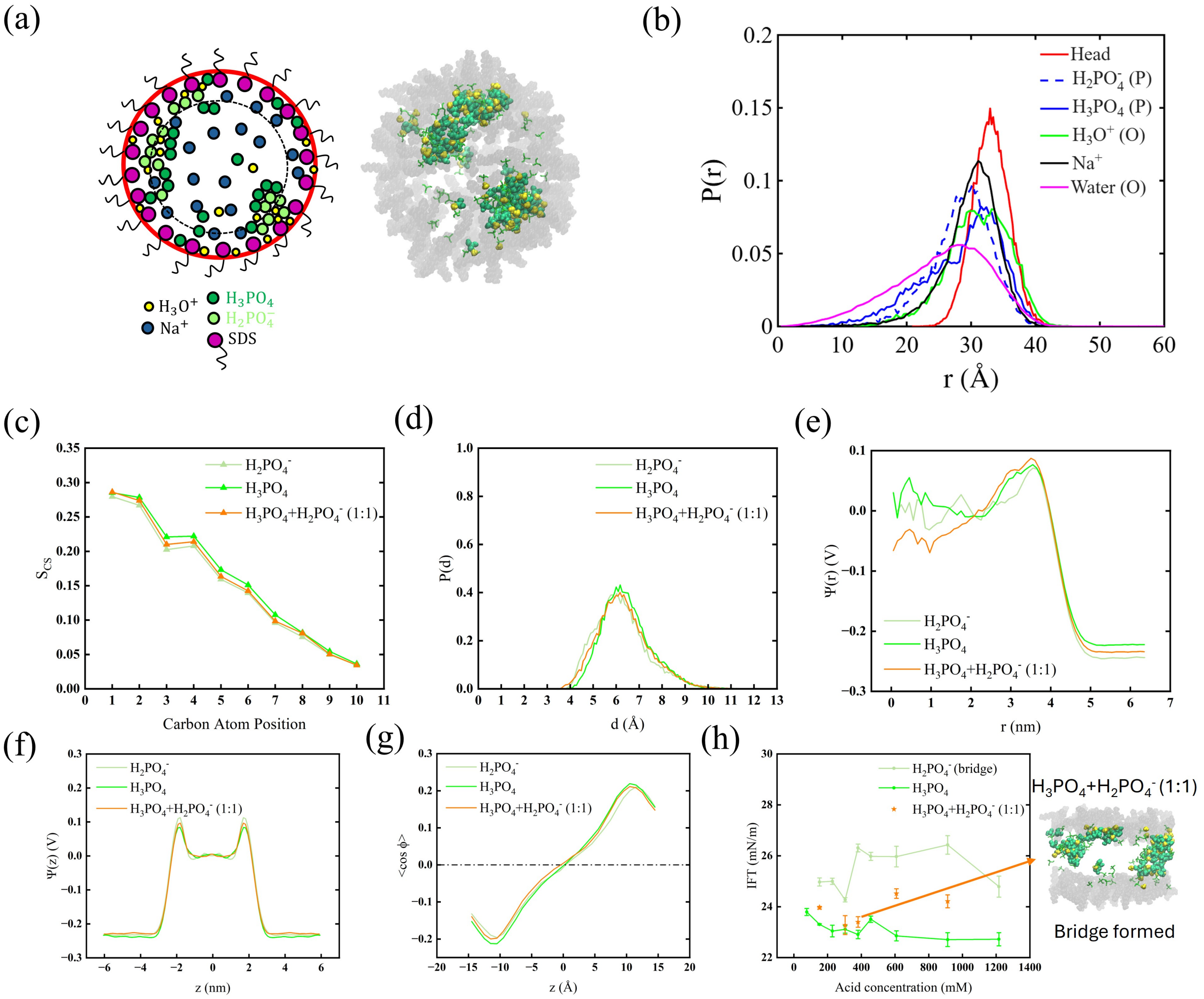

FIG. S17. (a) The schematic acidic species distribution inside the RM_I (left). The last snapshot of RM_I (right). The acid is in the mixture of dissociated H_3PO_4 and non-dissociated H_3PO_4 with a ratio of 1:1. The acid concentration is 158 mM. (b) Probability density distribution of the distance of the specific types of atoms from the COM for all SDS surfactants in the case of dissociated H_3PO_4 and non-dissociated H_3PO_4 (1:1). (c) represents S_{CS} for RM_I. (d) Probability density distribution of the nearest distance ($P(d)$) between each two sulfur atoms of head groups for RM_I. (e) and (f) Electrostatic potential of BI_I ($\psi(z)$) and RM_I ($\psi(r)$). (g) Water orientation of BI_I. (h) The interfacial tension (IFT) for BI_I. And $H_2PO_4^-$ are represented by dark green beads, H_3PO_4 are represented by bright green tubes, H_3O^+ are represented by yellow beads and SDS surfactants are shown in transparent black beads. Toluene and water are not shown for clarity.

SUPPLEMENTARY TABLES

TABLE S1. Coordinate-pair eccentricity (CPE) for RM_I and RM_{II} respectively.

System	Acid concentration	e_{ab}	e_{ac}
RM_I	No-acid	0.32±0.08	0.48±0.07
RM_I	158 mM HBF_4	0.30±0.07	0.44±0.06
RM_I	158 mM $HAuCl_4$	0.30±0.08	0.45±0.07
RM_I	158 mM H_3PO_4	0.31±0.08	0.45±0.07
RM_I	158 mM H_2SO_4	0.29±0.07	0.43±0.09
RM_I	395 mM HBF_4	0.28±0.09	0.43±0.06
RM_I	395 mM $HAuCl_4$	0.30±0.08	0.44±0.06
RM_I	395 mM H_3PO_4	0.28±0.07	0.43±0.06
RM_I	395 mM H_2SO_4	0.27±0.08	0.44±0.06
RM_{II}	No-acid	0.27± 0.07	0.40±0.06
RM_{II}	193 mM HBF_4	0.24±0.07	0.36±0.05
RM_{II}	193 mM $HAuCl_4$	0.26±0.07	0.37±0.06
RM_{II}	193 mM H_3PO_4	0.26±0.07	0.38±0.06
RM_{II}	193 mM H_2SO_4	0.27±0.07	0.38±0.06

TABLE S2. Hydrogen bonds (H-bonds) decay time constant.

System	Acid concentration	H-bond type	T_{HB}
RM_I	158 mM HBF_4	$Head^- \cdots H_3O^+$	241.2±30.6 ps
RM_I	158 mM $HAuCl_4$	$Head^- \cdots H_3O^+$	346.7±26.8 ps
RM_I	158 mM H_2SO_4	$Head^- \cdots H_3O^+$	326.1±39.9 ps
RM_I	395 mM HBF_4	$Head^- \cdots H_3O^+$	379.8±39.9 ps
RM_I	395 mM $HAuCl_4$	$Head^- \cdots H_3O^+$	810.0±106.8 ps
RM_I	395 mM H_2SO_4	$Head^- \cdots H_3O^+$	697.9±91.5 ps
RM_{II}	193 mM HBF_4	$Head^- \cdots H_3O^+$	223.6±20.8 ps
RM_{II}	193 mM $HAuCl_4$	$Head^- \cdots H_3O^+$	341.4±22.1 ps
RM_{II}	193 mM H_2SO_4	$Head^- \cdots H_3O^+$	323.6±29.9 ps
RM_I	158 mM H_3PO_4	$Head^- \cdots H_3PO_4$	210.1±26.0 ps
RM_I	158 mM H_2SO_4	$Head^- \cdots HSO_4^-$	184.6±14.4 ps
RM_I	395 mM H_3PO_4	$Head^- \cdots H_3PO_4$	262.2±22.1 ps
RM_I	395 mM H_2SO_4	$Head^- \cdots HSO_4^-$	431.6±60.6 ps
RM_I	158 mM H_3PO_4	$H_3PO_4 \cdots H_3PO_4$	460.7±64.0 ps
RM_I	158 mM H_2SO_4	$HSO_4^- \cdots HSO_4^-$	111.5±14.0 ps
RM_I	395 mM H_3PO_4	$H_3PO_4 \cdots H_3PO_4$	836.4±107.5 ps
RM_I	395 mM H_2SO_4	$HSO_4^- \cdots HSO_4^-$	315.3±16.3 ps

TABLE S3. Interfacial acid concentration of RMs and SDS bilayers. The unit is per molecule/nm^2.

System	c(SDS)	$c(H_3O^+/BF_4^-)$	$c(H_3O^+/AuCl_4^-)$	$c(H_3PO_4)$	$c(H_3O^+/HSO_4^-)$
RM_I	1.30	1.15/0.71	1.15/0.74	0.94	1.06/0.60
BI_I	1.11	1.15/0.75	1.16/0.74	0.89	1.02/0.61
RM_{II}	1.27	1.23/0.65	1.26/0.59	0.90	1.18/0.58
BI_{II}	1.14	1.08/0.56	1.09/0.59	0.80	0.97/0.46

[1] Sunaina, S. K. Mehta, A. K. Ganguli, and S. Vaidya, Small-angle X-ray scattering as an effective tool to understand the structure and rigidity of the reverse micelles with the variation of surfactant, J. Mol. Liq. **326**, 115302 (2021).

[2] S. Sunaina, V. Sethi, S. K. Mehta, A. K. Ganguli, and S. Vaidya, Correction: Understanding the role of co-surfactants in microemulsions on the growth of copper oxalate using SAXS, Phys. Chem. Chem. Phys. **21**, 17441 (2019).

[3] R. E. Ridley, H. Fathi-Kelly, J. P. Kelly, V. R. Vasquez, and O. A. Graeve, Predicting the size of salt-containing aqueous Na-AOT reverse micellar water-in-oil microemulsions with consideration for specific ion effects, J. Colloid Interface Sci. **586**, 830 (2021).

[4] R. E. Ridley, H. Fathi-Kelly, J. P. Kelly, V. R. Vasquez, and O. A. Graeve, Predicting Destabilization in Salt-Containing Aqueous Reverse Micellar Colloidal Systems, ACS Earth Space Chem. **5**, 2223 (2021).

[5] L. Abraham, T. Thomas, and M. Pichumani, Ionic amphiphile stabilized reverse micellar systems and their implications for nanoencapsulation, Colloids Surf., A **620**, 126591 (2021).

[6] C. Jian, M. R. Poopari, Q. Liu, N. Zerpa, H. Zeng, and T. Tang, Reduction of Water/Oil Interfacial Tension by Model Asphaltenes: The Governing Role of Surface Concentration, J. Phys. Chem. B **120**, 5646 (2016).

[7] Y. Lin, W. Tang, P. Xiao, J. Ma, X. Han, X. Xu, J. Luo, and S. Zhao, Synergistic Effect of Salt and Anionic Surfactants on Interfacial Tension Reduction: Insights from Molecular Dynamics Simulations, Langmuir **39**, 12392 (2023).

[8] K. R. Zürbes, E. Mani, and S. Bandyopadhyay, Synthesis of anisotropic gold nanoparticles in binary surfactant mixtures: a review on mechanisms of particle formation, RSC Adv. **15**, 4377 (2025).

[9] J. S. Hub, B. L. De Groot, and D. Van Der Spoel, g_wham—A Free Weighted Histogram Analysis Implementation Including Robust Error and Autocorrelation Estimates, J. Chem. Theory Comput. **6**, 3713 (2010).
